# The “Hard Problem of Consciousness” Arises from Human Psychology

**DOI:** 10.1162/opmi_a_00094

**Published:** 2023-08-11

**Authors:** Iris Berent

**Affiliations:** Northeastern University, Boston, MA, USA

**Keywords:** consciousness, the hard problem, intuitive psychology, dualism, essentialism

## Abstract

Consciousness presents a “hard problem” to scholars. At stake is how the physical body gives rise to subjective experience. Why consciousness is “hard”, however, is uncertain. One possibility is that the challenge arises from ontology—because consciousness is a special property/substance that is irreducible to the physical. Here, I show how the “hard problem” emerges from two intuitive biases that lie deep within human psychology: Essentialism and Dualism. To determine whether a subjective experience is transformative, people judge whether the experience pertains to one’s essence, and per Essentialism, one’s essence lies within one’s body. Psychological states that seem embodied (e.g., “color vision” ∼ eyes) can thus give rise to transformative experience. Per intuitive Dualism, however, the mind is distinct from the body, and epistemic states (knowledge and beliefs) seem particularly ethereal. It follows that conscious perception (e.g., “seeing color”) ought to seem more transformative than conscious knowledge (e.g., knowledge of how color vision works). Critically, the transformation arises precisely *because* the conscious perceptual experience seems readily embodied (rather than distinct from the physical body, as the ontological account suggests). In line with this proposal, five experiments show that, in laypeople’s view (a) experience is transformative only when it seems anchored in the human body; (b) gaining a transformative experience effects a bodily change; and (c) the magnitude of the transformation correlates with both (i) the perceived embodiment of that experience, and (ii) with Dualist intuitions, generally. These results cannot solve the ontological question of whether consciousness is distinct from the physical. But they do suggest that the roots of the “hard problem” are partly psychological.

## INTRODUCTION

Consciousness presents a “hard problem” to scholars (Chalmers, 1996). The “problem” is to explain how physical processes in the brain give rise to subjective experience. How does the brain make a blooming rose feel lush and sensuous and snow seem still and silent? How can electrochemical signals invoke sublime glory when we hear Bach’s music?

At hand is not simply the question of how conscious stimuli are encoded by the mind/brain (e.g., Dehaene, 2014; Dennett, 1991; Gazzaniga, 2018); this problem, by comparison, is “easy” (Chalmers, 1996). The real conundrum is how subjective experience emerges from the body: how the brain—a chunk of meat—produces a subjective “feel”. This is the “hard problem” of consciousness (Chalmers, 1996).

David Chalmers (Chalmers, 1996) suggests that such explanations are bound for failure: it is impossible to reduce a subjective phenomenal experience to the physical. This is not merely due to the cognitive limitations of humans or their current narrow understanding of physics or neuroscience. Rather, the problem arises from what experience *is* and how it relates to the physical world.

Our reality, according to Chalmers, exhibits Dualism—the properties of our subjective conscious experience (hereafter: “experience”) are distinct from the physical. For this reason, experience cannot be explained by physical brain processes. The hard problem, then, ultimately arises from *ontological* Dualism. And the implications of this proposal are wide reaching. The possibility that some natural states are irreducible to the physical challenges the scientific understanding of matter and poses principled limits on the scope of scientific explanation.

Here, I outline an alternative account for the “hard problem”. To explain why consciousness is difficult, there is no need to solve the problems of ontology—suffice it to examine human psychology. It is our *psyche* that renders minds ethereal, distinct from physical bodies. This intuitive Dualism, coupled with another psychological bias, Essentialism, can explain each of the arguments marshalled by Chalmers in support of the claim that the subjective and the physical are distinct, ontologically.

The possibility that the “hard problem” might be rooted in human psychology has not escaped Chalmers. In fact, Chalmers presents numerous principled arguments against this possibility (the so called “meta-problem”; Chalmers, 2018). But of course, the force of this critique can only be judged against concrete alternatives.

In what follows, I articulate a specific proposal of how the “hard problem” could potentially arise from within. I proceed to show that the psychological and ontological accounts generate opposite predictions. The experiments reported here exploit this divergence to adjudicate between these two competing explanations.

To be clear, my proposal does not speak to the question of whether bodies and minds are indeed separate. As noted, my concern is strictly with human psychology. But inasmuch as human psychology presents a simple alternative explanation for why consciousness seems irreducible to the physical, the force of the ontological argument is weakened. Quite possibly, then, the “hard problem” arises from within. What’s “hard” isn’t ontology; it’s human psychology.

### The Support for the Ontological Claim

To support the conclusion that consciousness is irreducible to the physical, Chalmers presents several scenarios whereby an agent’s conscious states seem to dissociate from physical properties—in line with the “hard problem”; the intuitions elicited by these cases are thus dubbed “problem intuitions”.

For the sake of expository clarity, here, I focus on one such case. I chose this example because it seems to produce particularly strong “problem” intuitions, and because a priori, it seems least amenable to explanation by intuitive psychology. Furthermore, as we will next see, the psychological and ontological accounts generate sharply opposite predictions for this example. Accordingly, this case study presents both the strongest challenge to the psychological proposal and the best opportunity to experimentally adjudicate between the two competing explanations; all these cases are discussed in the Supplementary Materials (SM).

The case in question is that of *Mary in the black and white room* (Jackson, 1982). Suppose that Mary, a vision neuroscientist, knows everything there is to know about the physical bases of color vision. Mary, however, lives in a black and white room, so she has never seen color. Suppose Mary were to step out of the black and white room and experience color for the first time. Has she gained something new?

Chalmers reasons that, had consciousness been fully explicable by physical facts, then Mary’s experience should not have conferred her with any novelty. But our intuitions are markedly different. We feel that Mary’s lack of subjective experience with color clearly deprives her of something significant. And thus, once she sees color for the first time, her understanding has been dramatically transformed. If so, Mary’s subjective experience is irreducible to physical facts.

For Chalmers, the perennial resistance of consciousness to physical explanation presents evidence regarding ontology: “the failure of logical supervenience directly implies that materialism is false: there are features of the world over and above the physical features” (Chalmers, 1996, p. 123). And while Chalmers’ argument is strictly pitched from explanation to ontology (rather than from ontology to explanation), it stands to reason that the two are linked (Chalmers, 2018): it is our dualist ontology that renders consciousness logically non-supervenient on the physical. In what follows, I thus refer to this proposal as the “ontological explanation”. I next show how these intuitions can be captured psychologically.

### The View From Intuitive Psychology

As we consider the vivid case of Mary and read Chalmers’ lucid writing (Chalmers, 1996), we are tempted to conclude that our strong gut reaction arises because, indeed, Mary’s newly gained experience is utterly removed from the physical, just as Chalmers suggests, and that these intuitions give us insight into how things really are, ontologically.

But as every magician knows too well, our intuitive explanation of our experiences can be illusory. And this could well be the case for the “consciousness magic”.

Yes, our transformative intuitions are strong, and yes, they concern the gap we perceive between the ethereal and the physical. But the psychological links we draw between the two may be quite different from what Chalmers implies.

To explain these differences, let us take a closer look at Mary’s case, as seen from the lens of the ontological account. First, Mary’s encounter with color principally confers her with new *knowledge*. Indeed, “insofar as it seems clear that when she sees red for the first time, Mary is *discovering* something about the way the world is, it seems clear that the knowledge she is gaining is knowledge of a fact” (Chalmers, 1996, p. 104). Second, Mary’s color experience is irreducible to the physical (“facts about the subjective experience of color vision are not entailed by the physical facts”, p. 103). Third, Mary’s color experience is irreducible to the physical because of the gap that exists between what’s physical and what isn’t.

To directly contrast Chalmers’ account with the psychological alternative and evaluate them experimentally, we will further need to consider how *readers* might react to what Mary knows and how things are. These psychological consequences constitute “linking hypotheses” that bridge the ontological account with the experimental investigation.

Chalmers himself does not say much about Mary’s reaction to her new experience (or the reader’s) and he doesn’t explicitly consider why such reaction arises. But he clearly implies that the experience is novel and significant (as without it, Mary would remain “entirely in the dark” about the experience of “red”, p. 10). Accordingly, I assume that readers consider Mary’s color experience *transformative*. This is the first linking hypothesis.

Since Chalmers further expects Mary’s case to elicit “problem intuitions”, and he considers those intuitions as evidence for what exists (ontology), he seems to assume that human intuitions are (at least) partly privy to what exists. And as noted, in his account, color experience confers Mary new knowledge because this experience goes beyond the physical facts. If readers are privy to how things are, ontologically, then they, too, might view Mary’s experience as non-physical, and this recognition could be at the root of their psychological reaction. Thus, people consider Mary’s experience transformative *because* they view it nonphysical; this is the second linking hypothesis. To reiterate, these two linking hypotheses go beyond Chalmers’ original claims.

1)  The ontological account of Mary’s case:
  a)  Mary’s color experience confers her new knowledge.
  b)  Mary’s color experience is irreducible to the physical.
  c)  Mary’s color experience is irreducible to the physical because of the gap that exists between what is physical and what is not.
  d)  Psychological linking hypotheses:
    i)  Mary’s color experience seems transformative.
    ii)  Mary’s color experience seems transformative because it seems non-physical.

Like the ontological account, the psychological account predicts that the strong transformation in Mary’s case arises from a gap between the physical and the non-physical (2(c)). But the specific nature of this gap and its causes differ sharply from what the ontological account suggests.

First, in the psychological account, it is Mary’s color experience that seems not only transformative but also strongly embodied; her previous knowledge (prior to seeing color), by comparison, seems relatively ethereal. In fact, the transformation arises precisely *because* Mary’s experience can be linked to her body, *more* so than her previous knowledge.

2)  The psychological account of Mary’s case:
  a)  Mary’s perceptual experience seems transformative and embodied.
  b)  The transformation, in Mary’s case, arises because her conscious perceptual experience seems *more* strongly embodied than her previous knowledge (which seems ethereal).
  c)  Mary’s experience is transformative because of the gap we perceive between what is physical and what is not.
  d)  The gap we perceive between the physical and the nonphysical arises from human psychology.
  e)  Mary’s encounter with color confers her new embodied experience.

From the psychological lens, then, Mary’s encounter with color confers primarily embodied perceptual experience, not some ethereal knowledge. And while, as noted, the experience critically arises from a gap between the physical and the ethereal, the source of the gap is squarely psychological, not ontological. Next, we turn to consider how this gap arises and how it yields the “problem intuitions”.

#### The Mind-Body Divide is Psychological.

In the ontological account, the tension we perceive between the physical and the nonphysical reflects reality. But, of course, there is no guarantee that our intuitions are veridical. The gap could be a fickle of our imagination, authored by the human mind.

A large literature has indeed documented an *intuitive Dualist* bias in laypeople—adults and children, in both Western participants (Berent, 2021a; Berent & Platt, 2021a, 2021c; Berent et al., 2021; Bering et al., 2005; Bloom, 1994; Cohen & Barrett, 2008; Forstmann & Burgmer, 2015; Forstmann et al., 2012; Heflick et al., 2015; Lane et al., 2016; Sandoboe & Berent, 2021; Slingerland & Chudek, 2011; Stanovich, 1989; Weisman et al., 2017) and members of small-scale societies (Astuti & Harris, 2008; Boyer, 2018; Chudek et al., 2018; Cohen et al., 2011; Watson-Jones et al., 2017; Weisman et al., 2021).

For example, when asked to predict which of a person’s traits would persist in the afterlife, after the demise of the body, people consider psychological traits, especially epistemic traits, as more likely to persist (Berent et al., 2022; Bering & Bjorklund, 2004). But when people are presented with a manipulation that targets the body (e.g., a replica of one’s body; a brain scan), here, psychological traits, especially, epistemic ones, seem the least likely to persist (Berent et al., 2021, 2022; Forstmann & Burgmer, 2015; Hood et al., 2012).

The fact that intuitions about bodies and minds *shift*, depending on whether the scenario targets the body or its demise, suggests that people consider the mind as relatively ethereal, distinct from the physical body. This is in line with intuitive Dualism.

##### *Mary’s Perceptual Experience is Embodied*.

Intuitive Dualism, as seen here, is a “soft” violable constraint. Dualism does not imply that people would necessarily posit a sharp dichotomy between the mental and the physical, or that all psychological traits ought to seem equally ethereal. Rather, Dualist intuitions are graded and relative.

Accordingly, when compared to the body, psychological traits seem ethereal (e.g., Bering & Bjorklund, 2004). But when compared against each other, some psychological traits seem more ethereal than others. “Seeing”, for instance, seems readily anchored in the body (in the eyes), whereas knowledge and beliefs, by comparison, seem disembodied (Berent, 2023; Berent et al., 2021, 2022).

Two aspects of Mary’s case render the comparison of mental states particularly salient (unlike all other problem intuitions, discussed in the SM). First, Mary’s case is framed as a contrast between two states—perception and knowledge. Second, the case invites to evaluate a change—hence, as how Mary’s newly gained color experience differs fundamentally from her previous knowledge.

The psychological analysis, then, predicts that Mary’s knowledge (in the black and white room) ought to seem particularly ethereal, *more* so than her perceptual experience—a prediction that stands in stark contrast to the ontological account. In fact, as we next see, from the psychological perspective, Mary’s conscious perceptions are transformative precisely *because* they are embodied. To explain how the transformation arises, we now need to consider the interaction of intuitive Dualism with a second psychological principle—Essentialism.

##### *Transformative Experiences Tap Into Our Embodied Essence*.

Some experiences are transformative. We can be moved to tears by a story, profoundly shaken by a piece of art, or transformed by a session of psychotherapy that has given us insights into our psyche. Mary’s first conscious encounter with color is likewise a transformative experience.

We seem to consider these experiences transformative because they tap into our core; they connect with who we *really* are “deep down”. And when people evaluate whether a *change* has fundamentally altered an agent—be it animal (e.g., a racoon painted like a skunk; Keil, 1986), or a person (e.g., a corrupt officer turned honest: Newman et al., 2014; see also Berent & Platt, 2021c; De Freitas & Cikara, 2018; De Freitas, Cikara, et al., 2017; De Freitas, Tobia, et al., 2017; De Freitas et al., 2018; Strohminger et al., 2017)—they invoke Essentialism.

Essentialism—the intuitive belief that living things possess an innate, immutable essence—guides reasoning about both biological kinds (Gelman, 2003; Gelman & Wellman, 1991; Keil, 1986; Medin & Ortony, 1989) and psychological phenomena (Gelman, 2004; Haslam et al., 2004), including consciousness. For example, Essentialism explains why a person in permanent vegetative state seems “more dead than the dead” (Gray et al., 2011). If people believe that one’s subjective experiences form part of one’s core, then a living person devoid of consciousness would seem uncanny indeed (Gray & Wegner, 2012).

Critically, there is evidence that this essence seems to reside in the person’s body (for review: Berent, 2021b). For example, when provided evidence that a psychological trait (e.g., depression) lies within the body (e.g., in the brain), people are more likely to essentialize the trait—to consider it as innate, immutable (Berent & Platt, 2021a, 2021b; Berent et al., 2021), and diagnostic of their “true self” (Berent & Platt, 2021c). Similarly, children align a person’s self with their eyes (Starmans & Bloom, 2012).

Summarizing, then, Mary’s case invites us to evaluate whether the change engendered by her experience is transformative. Transformative changes are ones that tap into one’s essence, and this essence, in turn, seems to reside in one’s body. So, to be transformative, Mary’s new experience ought to be readily anchored in her body. Color vision fits the bill. So, in the case of Mary’s color vision, we conclude that indeed, her experience is transformative.

**Figure 1.**
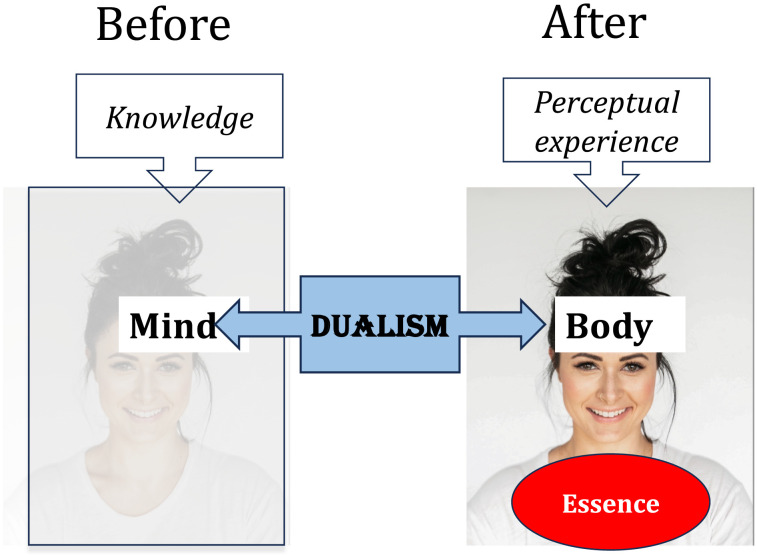
Knowledge vs. conscious experience of color vision.

Moving to intuitive Dualism, we can now explain why Mary’s conscious perception seems *more* transformative than her previous knowledge (of how color vision works). Per intuitive Dualism, knowledge seems ethereal. Knowledge, then, cannot possibly pertain to Mary’s embodied essence, so it isn’t transformative. Taken as a whole, then, Mary’s conscious perception is more likely to tap into her embodied essence, hence, it seems more transformative than ethereal knowledge (see (3); Figure 1).

3)  How intuitive Essentialism and Dualism give rise to the intuition that Mary’s experience is transformative.
  a)  Conscious perceptions are potentially transformative.
    i)  Transformative experiences tap into one’s embodied essence.
    ii)  Conscious perceptions are embodied.
    iii)  Conscious perceptions can be transformative.
  b)  Conscious knowledge cannot be transformative.
    i)  Transformative experiences tap into one’s embodied essence.
    ii)  Per intuitive Dualism, knowledge is ethereal.
    iii)  Conscious knowledge cannot be transformative.

### Conclusions and Predictions

The ontological account asserts that Mary’s experience is transformative because her experience seems irreducible to the physical. And we arrive at this conclusion because of how things *are*—because experience is ontologically distinct from the physical.

The psychological analysis outlined here agrees that, indeed, people do contrast the physical and the nonphysical. But the transformative force of consciousness arises not because it seems irreducible to the physical body, but precisely because it seems readily embodied (more so than her previous knowledge, which seems ethereal). And all these intuitions arise not from ontology but from two psychological biases: Dualism and Essentialism.

Not only can the psychological account explain why Mary’s case generates such an “aha” moment, but it can further explain why, in the consciousness literature, it is typically perceptual, not epistemic experiences that seem paradigmatic of consciousness, generally.

From the ontological perspective, this fact is mysterious. If consciousness is significant because it is distinct from the physical, and the human mind is privy to this fact, then consciousness ought to seem uniformly transformative, regardless of whether the subjective experience arises from perception or from knowledge. The psychological account, by contrast, predicts this fact. It also generates a number of empirically testable predictions with respect to four key questions.

1. *Do all experiences seem uniformly transformative*? The psychological account predicts that they don’t—conscious perceptions seem more transformative than conscious knowledge. Mary’s case, however, does not allow us to test this prediction as it only features a single experience (perception). Accordingly, Experiments 1–5 systematically contrast experiences that concern perception vs. knowledge.

2. *Do transformative experience seem more readily anchored in the physical body* (as the psychological account predicts) *or less so* (as predicted the ontological account)? Experiments 1–5 examine this question by having participants evaluate whether the conscious mental state in question is transformative, and whether it is likely to “show up” in the brain.

We consider the “brain scan” as evidence for embodiment intuitions because (a) the brain forms part of the body, and (b) past research shows that intuitions about the propensity of psychological traits to “show up” in the brain agree with other measures of embodiment and disembodiment (body replica and afterlife, respectively, Berent, 2023; Berent & Platt, 2021b; Berent et al., 2021, 2022; Sandoboe & Berent, 2021). Specifically, knowledge, in these studies, is considered less likely to show up in both the brain and a body replica (Berent & Platt, 2021b; Berent et al., 2021), but more likely to persist in the afterlife (Berent, 2023; Berent et al., 2021, 2022). Thus, “in the brain” judgments likely tap into people’s embodiment intuitions. Of interest is whether experiences seem more likely to show up in the brain (as the psychological account); such that (i) transformative experiences appear *more* embodied; and (ii) the sense of transformation and embodiment correlate *positively*. Experiments 1–2 evaluate this question.

3. *Does gaining transformative experience seem to cause bodily changes*? The psychological account claims that it does. Transformative experiences pertain to one’s embodied essence (more so than an unconscious experience, for instance). Accordingly, gaining an experience ought to cause a detectible bodily change. These predictions stand in stark contrast to the claim that consciousness is irreducible to the physical. Experiment 3 evaluates this question; Experiment 4 does the same and further secures the conclusion that “in the brain” intuitions indeed reflect embodiment intuitions, linked to intuitive Dualism.

4. *Who can have transformative experience*? If transformative experiences seem to tap into our essence, and if essence chiefly defines living things (Gelman & Wellman, 1991; Keil, 1986), then transformative experience ought to be seen as falling within the purview of living things (e.g., humans), but perhaps not nonhuman AI. Experiment 5 tests this prediction.

## CONSCIOUS EXPERIENCES AREN’T UNIFORMLY TRANSFORMATIVE: EXPERIMENT 1

Experiment 1 examines whether all subjective experiences seem transformative, or whether perceptual experiences seem particularly amenable to yield transformation. To this end, we contrast two varieties of experiences—a conscious *perceptual* experience of color vision and conscious *knowledge*. For each such experience, participants evaluated its transformative value and its anchoring in the body.

The conscious perceptual experience is depicted in the case of Mary in the black-and-white room (as described above). Briefly, Mary is a neuroscience expert who knows all about the physical basis of color vision, yet she has never seen color; her new conscious perceptual experience arises as she sees a red rose for the first time.

To depict conscious knowledge, participants are introduced to Jack—a professional billiards player. Jack has perfect “gut” intuitions of how balls move. In particular, he can superbly predict their velocity and trajectory, and how their speed varies depending on the friction with the table. But Jack has never heard the terms “momentum” or Newtonian physics. Jack then takes a crash course in Physics, where he learns the laws of motion as they apply to launching billiard balls. Jack can now describe these laws perfectly, and he is consciously aware of this knowledge.

For each protagonist, participants respond to three questions. The first question is how transformative is the newly gained experience. The next two questions evaluate the embodiment of the protagonist’s mental states—both before and after gaining the experience.

To evaluate the initial state, participants are asked to imagine that each protagonist has an identical twin who does not possess their expertise—Mary’s twin sister knows nothing about color vision, whereas Jack’s twin knows nothing about billiard—and to suppose both twins undergo a brain scan. Participants are asked to evaluate how different the two scans would look—this is a measure of the embodiment of the protagonists’ expertise before gaining the experience. To evaluate the embodiment of the experience, participants are then asked to compare the brain scan of each expert (Mary/Jack) before and after gaining the experience.

The psychological account predicts that Mary’s conscious perceptual experience ought to seem more (a) transformative and (b) embodied than Jack’s conscious knowledge. Moreover, (c) the “transformative” and “embodiment” ratings of these experiences ought to correlate.

### Methods

#### Participants.

Experiment 1 was assigned to 30 participants.

In this and all subsequent experiments, participants were Prolific workers; they were all adult, native English speakers, who self-identified as having no neurological or language disorders and had no known diagnoses of autism. Their characteristics are summarized in Tables S1–S2.

Sample size in this and all subsequent experiments was based on pilot research, suggesting that the selected sample size was sufficient to attain a power of 80% at the alpha level of .05.

In this and all analysis, outliers (participants whose responses fell 2.5 *SD* beyond the cell mean) were removed from all analyses.

Two participants were excluded from Experiment 1. Additionally, the data of three participants were incomplete. Thus, the analysis of Experiment 1 was based on 25 participants.

#### Materials.

Materials and procedures for this and all subsequent experiments were reviewed and approved by the Institutional Review Board at Northeastern University. All participants signed (electronically) an informed consent form.

Experiments 1 contrasted the cases of Mary in the black and white room and Jack. As noted, Mary underwent a change, leading to the acquisition of a new experience. Prior to that event, Mary, a renowned neuroscientist of color vision, had an explicit understanding of color vision, but no first-hand experience seeing color; her new experience consisted of the experience of color vision (seeing a red rose for the first time).

Jack, by contrast, is a billiards expert who likewise acquired a new experience. Prior to that experience, Jack had a tacit understanding of how balls move, as he was an expert billiards player; his new experience was the acquisition of conscious knowledge of the physical laws of motion.

For each protagonist, participants answered three questions: (a) how transformative is the new experience? (b) how likely is the protagonist’s initial state (prior to the experience) to show up in a brain scan (i.e., would their scan differ from a twin sibling’s scan who lacked that experience)? And (c) how likely is the new experience to show up in a brain scan (i.e., would their brain scan after the experience differ from before)?

In this and all subsequent experiments, ratings were given on a 1-7 scale, and (unless noted otherwise) the order of the two vignettes was randomized. All materials are presented in Supporting Information, Appendix I.

### Results and Discussion

Participants’ ratings were submitted to three analyses. First, we compared the transformative impact of conscious perception vs. knowledge. Second, we examined the embodiment of the protagonist’s mental state before vs. after gaining these experiences. Third, we correlated the “transformative” and “embodiment” ratings of the conscious states. All analyses were performed in JASP (JASP Team, 2022), and figures were generated therein, whereby SE are normalized standard errors (Morey, 2008).

#### Is Experience Transformative?

**Figure 2.**
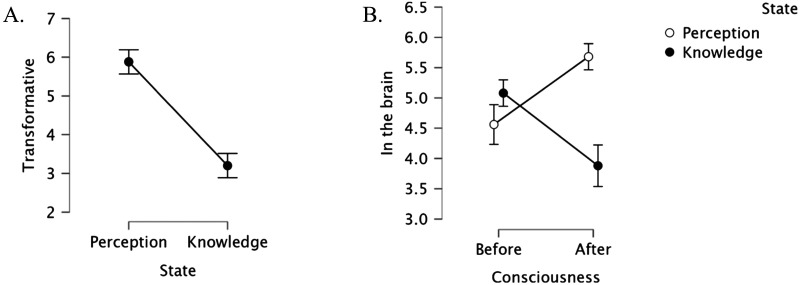
The transformative effect of conscious experiences (A) and their embodiment (in the brain; B). Error bars are normalized *SE*.

The transformative ratings of the two experiences—color perception vs. knowledge—were compared via a paired t-test. Results (see Figure 2A) showed that the conscious perceptual experience (seeing color) was considered more transformative than the conscious knowledge (of physics: *t*(24) = 6.06, *p* < .001; *d* = 1.212).

To evaluate whether participants indeed viewed each such experience as transformative, the two means were next compared against the scale’s neutral midpoint (4, “cannot tell”). Results showed that Mary’s conscious perceptual experience was clearly considered transformative (*t*(24) = 7.61, *p* < .001, *d* = 1.522), whereas Jack’s conscious knowledge was decidedly rated as non-transformative (i.e., below the scale’s midpoint: *t*(24) = −2.38, *p* = .03, *d* = −0.475).

#### Embodiment.

We next evaluated the embodiment of the protagonists’ mental states “before” and “after” gaining that conscious state via 2 State (Vision vs. Knowledge) × 2 Consciousness (Before/After) within-subjects ANOVA.

Results (Figure 2B) yielded a reliable main effect of State (*F*(1, 24) = 5.26, *p* = .03, *η*_*p*_^2^ = 0.18), as Mary’s was considered more likely to show up in the brain than Jack’s. The main effect of Consciousness was not significant (*F* < 1), but, critically, State and Consciousness interacted (*F*(1, 24) = 15.75, *p* < .001, *η*_*p*_^2^ = 0.396).

A test of the simple main effects showed that, in Mary’s case, her conscious perception (after) was considered more likely to show up in her brain than her knowledge (before): (*F*(1, 24) = 8.12, *p* < .01). Jack’s case showed the opposite: his conscious knowledge was considered less likely to manifest in the brain than his tacit perceptual experience (*F*(1, 27) = 8.47, *p* < .009).

Furthermore, when judged in absolute terms (compared to the scale’s “neutral” midpoint), Mary’s conscious perception seemed embodied (*t*(24) = 8.16, *p* < .001, *d* = 1.632), whereas her knowledge was not (*t*(24) = 1.57, *p* > .13, *d* = 0.314). Jack’s conscious understanding of physics, by contrast, was not considered embodied (*t* < 1), whereas his tacit understanding was seen as embodied (*t*(24) = 4.55, *p* < .001, *d* = 0.91), possibly because this implicit awareness was construed as a motor skill (e.g., manipulating the billiard balls) involving his limbs.

#### Does Consciousness Depend on Embodiment?

To evaluate whether the transformative value of an experience is linked to its embodiment, we next correlated these two ratings. The correlations were positive and they were marginally significant for Mary’s perception (*r*(24) = .362, *p* = .08) and significant for Jack’s knowledge (*r*(24) = .601, *p* < .001).

Summarizing, Experiment 1 yielded three main conclusions. First, conscious states are not uniformly transformative: Mary’s conscious perceptual experience seems transformative, but Jack’s knowledge does not. Second, Mary’s transformative experience of color vision is considered *more* likely to show up in her brain than her previous knowledge. Third, intuitions of transformation and embodiment tend to correlate.

Neither of these findings is expected by the ontological account, and the greater embodiment of conscious visual experience is plainly contradictory to this proposal. However, Experiment 1 cannot establish why perception and knowledge differ on their transformative power. Indeed, the cases of Mary and Jack are limited, inasmuch as they differ on multiple levels, so it is unclear that their observed differences concern perception vs. knowledge, specifically. Experiment 2 evaluates this question.

## TRANSFORMATIVE CONSCIOUS EXPERIENCES ARE EMBODIED: EXPERIMENT 2

To further evaluate the contrast between conscious perception and knowledge, Experiment 2 contrasts them in a single domain—color vision. To this end, the case of Mary (from Experiment 1) is compared with Susan—a renowned artist who specializes in vivid renditions of red roses. Although Susan has perfect “gut” intuitions about how to mix colors to create the perfect red hue and how to manipulate color so it strikes the human viewers, Susan has no conscious knowledge of how colors work—she has never studied these matters explicitly; her understanding is purely tacit. Suppose Susan takes a crash course in the science of color vision, where she learns about the principles of color chemistry, optics and the psychology of color vision, and she now becomes consciously aware of these principles.

For both protagonists, participants responded to three questions (as in Experiment 1): (a) how transformative is the experience? (b) how likely is the protagonist’s “before” state to “show up” in a brain scan (i.e., would it differ from the scan of her twin sister, who has no knowledge/artistic grasp of color)? And (c) how likely is the new experience to “show up” in the brain (relative to the “before” state)? To avoid carry-over effects between the two similar vignettes, they were each presented to a distinct group of participants (i.e., between subjects); State (before/after) was manipulated within subjects.

### Methods

#### Participants.

Experiment 2 was assigned to two groups of participants (*N* = 30 each). One participant was an outlier, and data from two participants were incomplete. Thus, the analyses of Experiment 2 were based on 57 participants. Sample size in this (between-participants) design was set by arbitrarily doubling the sample of Experiment 1.

#### Materials.

Experiment 2 contrasted Mary (the vision scientist in the black and white room, as in Experiment 1) and Susan, a renowned artist who specialized in painting red roses. Prior to acquiring her new experience, Susan had an intuitive “feel” of how color works, and she subsequently acquired conscious knowledge of the principles of color chemistry, optics and neuroscience. The two vignettes were assigned to two distinct groups of participants. Participants answered the same three questions as in Experiment 1.

### Results and Discussion

#### Is Consciousness Transformative?

**Figure 3.**
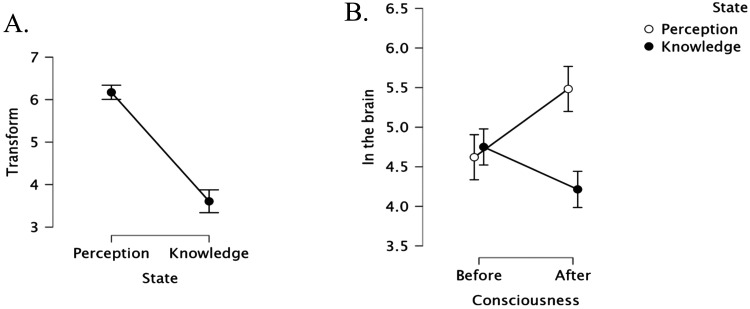
The transformative effect of conscious experiences (A) and their embodiment (in the brain; B). Error bars are normalized *SE*.

As in Experiment 1, gaining a conscious perceptual experience was considered more transformative than gaining conscious knowledge (*t*(55) = 8.19, *p* < .001, *d* = 2.171; see Figure 3A). Additionally, when compared to the “neutral” midpoint of the rating scale, only the perceptual experience was transformative (*t*(29) = 13.62, *p* < .001, *d* = 2.486) whereas conscious knowledge was not (*t*(28) = −1.51, *p* > .14, *d* = −0.281).

#### Embodiment.

Responses to the “in the brain” question (see Figure 3B) were submitted to a 2 State (Perception vs. Knowledge) × 2 Consciousness (before vs. After) ANOVA. Results yielded a reliable interaction of Consciousness × State (*F*(1, 55) = 7.27, *p* < .01, *η*_*p*_^2^ = 0.117). The main effects of State (*F*(1, 55) = 2.56, *p* > .11, *η*_*p*_^2^ = 0.044) and Consciousness (*F* < 1) were not significant.

A test of the simple main effects of consciousness (before vs. after the experience) indicated that Mary’s perceptual experience was considered more embodied than her previous state (*F*(1, 55) = 4.59, *p* = .04), whereas Susan’s conscious knowledge was not (*F*(1, 55) = 2.75, *p* > .11). Additionally, comparing the states of the two agents *after* the experience, Mary’s conscious perception seemed more embodied than Susan’s conscious knowledge (*F*(1, 55) = 10.51, *p* < .002), whereas *before* the experience, the states of the two agents did not differ (*F* < 1).

Moreover, when compared against the scale’s “neutral” midpoint, Mary’s perceptual experience was considered embodied (*t*(29) = 5.99, *p* < .001, *d* = 1.094), whereas Susan’s conscious knowledge was not (*t* < 1).

#### Correlations.

A final analysis examined whether the “transformative value” and “embodiment” of experiences correlate. This was indeed in the case for Mary, whose perceptual experience seemed transformative (*r*(27) = .483, *p* < .007). No such correlation emerged for Susan’s conscious knowledge (which was considered non-transformative: *r*(24) = −0.027, *p* > .88).

Thus, Experiment 2 reaffirms that Mary’s conscious perceptual experience is both transformative and embodied, and these two ratings correlate. Critically, this was the case even when both experiences—perception and knowledge—were quite similar, as they both concerned color vision.

Still, the results of Experiments 1–2 do not make it clear whether the greater embodiment of Mary’s perceptual experience arises from consciousness, specifically. And indeed, when Mary gains her conscious perceptual experience, she also engages in a sensory process that involves her eyes. So perhaps it’s this sensory experience, rather than consciousness, that elicits the embodiment intuitions. Does a transformative experience, then, seem *sufficient* to effect change in the body (even when sensory experience is controlled)? Experiment 3 addresses this question.

## GAINING A CONSCIOUS EXPERIENCE CAUSES BODILY CHANGES: EXPERIMENT 3

To directly evaluate whether, in laypeople’s view, gaining an experience causes changes in the body, Experiment 3 further evaluates Mary’s case. Participants are told that John, a fellow scientist, wishes to examine how Mary’s first encounter with color will affect her brain. To this end, John considers two fMRI experiments.

Each such experiment presents Mary with an image of a red rose. In one experiment, however, the rose image is flashed subliminally, so that Mary is not consciously aware of what she had seen (if asked to report what she saw, Mary would have said “nothing”); still, past research has shown that the image is nonetheless perceived, as immediately after seeing it, the word *rose* is more readily recognized. In the second condition, the image is clearly visible (if asked, Mary would report seeing it); hence, in this experiment, Mary would clearly gain a conscious perceptual experience of color.

For each thought experiment, participants respond to two questions. First, would seeing the red rose “show up” in Mary’s brain? Second, how transformative is Mary’s first encounter with color?

Of interest is whether Mary’s experience would seem more embodied and transformative than the subliminal experience, even when both involve “seeing”. Thus, this experiment examines whether a transformative color experience is considered sufficient to effect change in the body.

### Methods

#### Participants.

Experiment 3 was assigned to 30 participants. One participant was removed for being an outlier. Thus, the analysis of Experiment 3 was based on 29 participants.

#### Materials.

Experiment 3 featured Mary (as in Experiment 1) as she acquired her first experience seeing a red rose—either subliminally and subconsciously, or consciously. Participants evaluated how likely each experience is to “show up” in Mary’s brain, and how transformative the experience is.

### Results and Discussion

**Figure 4.**
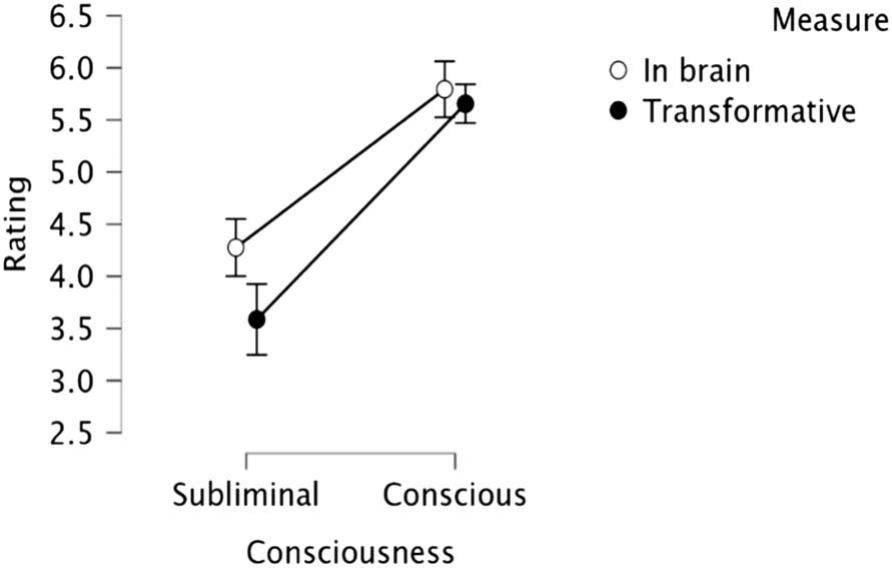
The transformative effect of conscious experiences and their embodiment (in the brain). Error bars are normalized *SE*.

Paired sample tests showed that participants considered Mary’s experience as more transformative compared to the unconscious subliminal experience (*t*(28) = 5.68, *p* < .001, *d* = 1.054; Figure 4). Critically, the conscious experience was also seen as more likely to show up in Mary’s brain (relative to the subliminal condition: *t*(28) = 4.21, *p* < .001, *d* = 0.783).

Furthermore, when compared to the scales’ “neutral” midpoint, the conscious experience was considered both transformative (*t*(28) = 7.80, *p* < .001, *d* = 1.45) and likely to “show up” in the brain (*t*(28) = 6.90, *p* < .001, *d* = 1.282). This, however, was not the case for the subliminal experience (both *p* > .21).

Finally, the correlation between the “transformative” and “in the brain” ratings was significant only in the conscious (*r*(24) = .513, *p* = .004), but not the subliminal experience (*r*(24) = .171, *p* > .37).

Thus, gaining consciousness renders a perceptual experience more embodied, even when compared to a subliminal experience that confers precisely the same sensation.

## EXPERIMENT 4

Experiment 4 has three goals. First, we seek to replicate the critical findings (of Experiment 3) that (a) Mary’s transformative experience seems more likely to “show up” in the brain, and (b) intuitions concerning whether the experience is “transformative” and “in the brain” are linked. Second, we wish to further demonstrate that the “brain” task indeed gauges embodiment. Third, we seek to probe whether intuitions regarding Mary’s case are linked to intuitive Dualism.

To this end, Experiment 4 administers two sets of tasks. The first set is identical to Experiment 3, and thus, we expect the conclusions of Experiment 3 to reemerge. The second set of tasks gauges participants’ intuitions about bodies and minds. Here, we present participants with a set of 80 traits, half epistemic and half non-epistemic; participants are asked to evaluate whether these traits would emerge in two situations—a body replica (a test of embodiment) and the afterlife (a test of disembodiment).

We expect that responses to epistemic traits to *shift* across the two scenarios. Thus, epistemic traits should be considered less likely to emerge in the replica, but more likely to emerge in the afterlife (compared to non-epistemic traits). The critical question is whether these Dualist intuitions are linked to intuitions concerning Mary’s case.

If the “in the brain” judgments gauge embodiment, then “in the brain” ratings ought to correlate positively with “replication” responses, especially for non-epistemic traits—the ones considered most embodied (e.g., Berent et al., 2022). Critically, if the sense of transformation (in Mary’s case) arises from embodiment intuitions, courtesy of Dualism, then participants who exhibit strong embodiment intuitions in the Dualist task ought to consider Mary’s perceptual experiences as more transformative.

### Methods

#### Participants.

Because our interest here was in individual differences in Dualism, we arbitrarily doubled the number of participants to 60. Four participants were identified as outliers and removed from all analyses.

#### Materials.

The experiment had two parts. The first part was identical to Experiment 3, and it elicited intuitions about Mary’s subliminal and experiences.

The second part of the experiment evaluated intuitive Dualism. To this end, participants were given two tasks (counterbalanced for order): body replication and afterlife. Each list featured 40 psychological traits (half epistemic, half non-epistemic; randomized for order). The replication task asked participants to evaluate whether these traits would emerge in a replica of a donor’s body; the afterlife task asked whether these traits would emerge in the afterlife. Participants were instructed to assume the afterlife exists—their task was to evaluate whether a given trait is likely to persist. Responses to the two Dualism tasks were binary (yes/no). Across the two tasks, each participant was assigned 80 traits. Because of a programming error, however, one of those traits (Seeing objects with one’s eyes) was not displayed.

### Results and Discussion

#### Mary’s Case.

**Figure 5.**
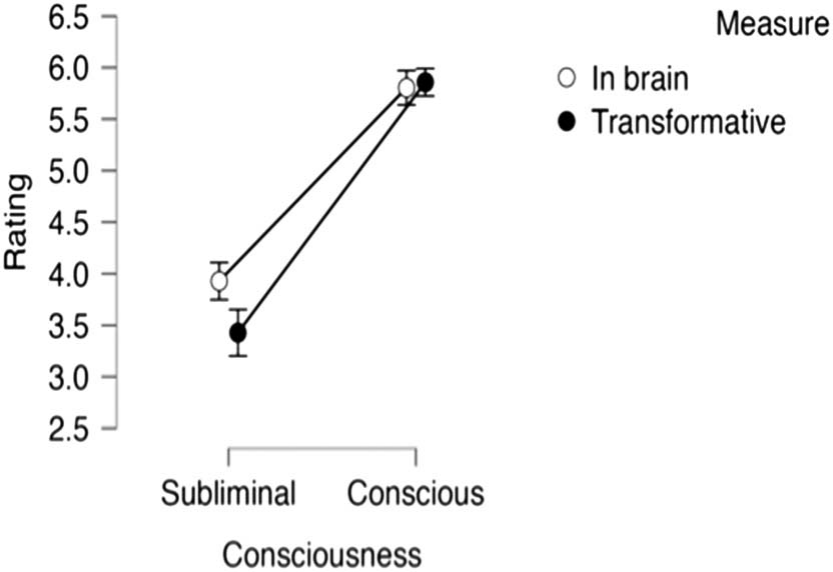
The transformative effect of conscious experiences and their embodiment (in the brain). Error bars are normalized *SE*.

As in Experiment 3, participants considered Mary’s conscious perceptions more transformative than her subliminal ones (*t*(55) = 9.26, *p* < .001, Cohen *d* = 1.24; see Figure 5). Moreover, Mary’s conscious perceptions also seemed more likely to “show up” in her brain (*t*(55) = 7.93, *p* < .001, Cohen *d* = 1.06).

Additionally, when compared against the scale’s neutral midpoint, Mary’s conscious perceptions seemed to “show up” in her brain (*t*(55) = 9.59, *p* < .001, Cohen *d* = 1.28) and they were considered “transformative”(*t*(55) = 11.46, *p* < .001, Cohen *d* = 153). In contrast, participants were unsure whether Mary’s subliminal perception would “show up” in the brain, as the mean did not significantly differ from the “neutral” midpoint (*t* < 1). Moreover, participants considered subliminal perception as squarely non-transformative (i.e., significantly below the “neutral” midpoint: (*t*(55) = 2.46, *p* = .02, Cohen *d* = 0.33).

Thus, only conscious perception seems transformative and “in the brain”. In fact, these two judgments (“transformative” and “in the brain”) correlated significantly in the conscious condition (*r*(54) = 633, *p* < .001; for the subliminal condition: *r*(54) = .241, *p* = .08). These results are unexpected by the ontological explanation. We next examine whether these intuitions are linked to intuitive Dualism.

#### Dualism.

To address this question, we first examined whether participants in this experiment veer towards Dualism. If they do, then they should consider epistemic traits as relatively disembodied—as more likely to emerge in the afterlife, and less so in the body replica.

**Figure 6.**
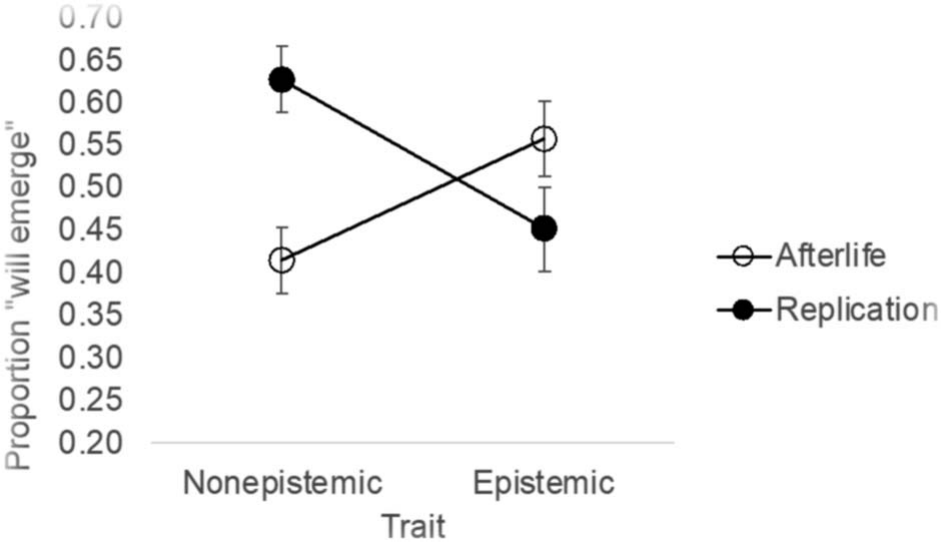
Dualist intuitions in the replication and afterlife tasks. Error bars are *SE*.

An inspection of the means (Figure 6) suggested that was indeed the case, and this conclusion was confirmed by a mixed-effect logistic regression model (trait * task + (trait * task | subject) + (1 | item)). The task * trait interaction was significant (*β* = 2.75, *SE* = 0.30, *z* = 9.04, *p* < .001).

A separate analysis of the two tasks showed that, in the replication task, epistemic traits were considered less likely to emerge than non-epistemic traits (*β* = 1.74, *SE* = 0.49, *z* = 3.56, *p* < .001), whereas the afterlife task yielded the converse—epistemic traits were considered more likely to emerge (*β* = −1.65, *SE* = 0.43, *z* = −3.86, *p* < .001). This confirms that people are indeed intuitive Dualist—they consider epistemic traits as relatively disembodied.

#### Task Comparison.

Having established that as a group, these participants exhibit intuitive Dualism, we next asked whether individual differences in Dualism can explain intuitions about Mary’s case.

The ontological account, recall, predicts that conscious states ought to seem distinct from the physical, contrary to our “in the brain” findings. But intuitions about the brain could potentially arise from factors that are unrelated to embodiment (e.g., knowledge of neuroscience). Accordingly, our first goal was to establish that “in the brain” responses indeed reflect embodiment intuitions; we next moved to evaluate the link between Dualist intuitions and “problem intuition” in Mary’s case.

#### “Brain” vs. “Replication” Intuitions.

We first examined whether “in the brain” responses reflect embodiment intuitions. If they do, then participants who believe that Mary’s perceptual experience—a non-epistemic state—resides “in her brain” ought to believe the same for other non-epistemic psychological traits. Accordingly, these participants should consider non-epistemic traits as likely to emerge in the body-replica.

A correlational analysis (Table 1) indeed showed that “in the brain” intuitions towards Mary’s conscious perception correlated with “replication” intuitions for non-epistemic traits. There were no significant correlations with epistemic traits—this is only expected if epistemic traits seem disembodied. Likewise, replication intuitions did not correlate with intuitions concerning Mary’s subliminal percepts, possibly, because those, too, seemed unlikely to register in her brain.

**Table 1.** The correlation between Dualist intuitions and responses to Mary’s case.

***Task***	***Mary’s case***				
	***Trait***	**In Brain**		**Transformative**	
		***Subliminal***	***Conscious***	***Subliminal***	***Conscious***
Replication	Non-epistemic	−.011	***.355*** **	−.170	.323*
	Epistemic	−.048	.206	−.095	.179
Afterlife	Non-epistemic	.076	**.336** *	.039	.158
	Epistemic	***.239*** *	***.331*** *	.093	.159

*Note*. Significance correlations are boldened.

* *p* < .05.

** *p* < .01.

However, “in the brain” responses also correlated with “afterlife” responses. Unlike the correlations for the “body replication” task, these positive “afterlife” correlations are clearly inexplicable by embodiment intuitions. These extraneous correlations suggest that the task-correlations are partly shaped by factors are unrelated to embodiment—possibly a response bias (towards “yes”/high rating).

To demonstrate that the link between “in the brain” and “replication” responses arises from embodiment (and not from response bias), we next submitted these results to a stepwise regression analysis (with forced entry of predictors). Steps 1–4 incrementally forced into the models the (1) afterlife responses to non-epistemic traits; (2) afterlife response to epistemic traits; (3) “in the brain” responses to Mary’s subliminal perception, and (4) replication responses to epistemic traits (these were the last predictors for steps 1-4, respectively). In the fifth model, all four steps were entered first; replication response to non-epistemic states was forced in last. Results (Table 2) showed that the effect of non-epistemic states remained significant after controlling for all other factors.

**Table 2.** The results of a stepwise linear regression analysis of the “in the brain” responses.

**Model**	***R***	***R* ^2^**	**Adjusted *R*^2^**	***SE* of the Estimate**	***Change Statistics***			
					***R*^2^ Change**	***F* Change**	***df***	**Sig. *F* Change**
1	.336^a^	0.113	0.097	1.337	0.113	6.879	1, 54	0.011
2	.355^b^	0.126	0.093	1.34	0.013	0.78	1, 53	0.381
3	.471^c^	0.222	0.177	1.276	0.096	6.449	1, 52	0.014
4	.514^d^	0.265	0.207	1.253	0.042	2.931	1, 51	0.093
5	.597^e^	0.356	0.292	1.184	0.092	7.131	1, 50	0.01

a Predictors: Non-epistemic (afterlife).

b Predictors: Non-epistemic (afterlife), Epistemic (afterlife).

c Predictors: Non-epistemic (afterlife), Epistemic (afterlife), Subliminal (brain).

d Predictors: Non-epistemic (afterlife), Epistemic (afterlife), Subliminal (brain), Epistemic (replication).

e Predictors: Non-epistemic (afterlife), Epistemic (afterlife), Subliminal (brain), Epistemic (replication), Non-epistemic (replication).

These results confirm that intuitions regarding the propensity of Mary’s perceptual experience to manifest in the brain indeed reflect the view of this experience as embodied, in line with past research (Berent, 2023; Berent & Platt, 2021b; Berent et al., 2021, 2022; Sandoboe & Berent, 2021).

#### Dualism vs. Consciousness Intuitions.

We next examined whether the “transformative” responses (for Mary’s case) are linked to intuitive Dualism. If they are, then participants who show strong transformative intuitions (in Mary’s case) should also show strong Dualist intuitions, especially with respect to embodiment—the putative cause of the transformation. Accordingly, participants who consider Mary’s perceptual (i.e., non-epistemic) experience as highly transformative ought to consider other non-epistemic traits as highly embodied—as likely to emerge in the body-replication task. Critically, if this association arises from intuitive Dualism, then the link ought to be *selective*—it should only emerge for non-epistemic traits (the ones seen as embodied), but not for epistemic traits (those that, in the Dualist eyes, aren’t embodied). And it should only apply for replication, but not afterlife intuitions.

Results (Table 1) support the psychological account. Participants who considered Mary’s experience as transformative tended to view non-epistemic psychological traits as embodied (as likely to manifest in the body-replica). And as expected, “transformative” intuitions concerning Mary’s conscious experience did not significantly correlate with replication response to epistemic traits, nor did they correlate with afterlife intuitions. Likewise, the replication and afterlife responses did not correlate with “transformative” intuitions in the subliminal condition. This selectivity is in line with intuitive Dualism.

Summarizing, the results of Experiment 4 confirm that (i) participants consider Mary’s conscious perception as more transformative, and as *more* likely to manifest in the brain; (ii) these two judgments correlate; and (iii) the “in the brain” task gauges embodiment. Altogether, then, these results suggest that transformative experiences seem *more* likely to manifest in the physical body.

There were also some indications that responses to Mary’s experiences were linked to intuitive Dualism. First, this group of participants exhibited intuitive Dualism. Moreover, participants who considered Mary’s experiences transformative also tended to view psychological traits as embodied (in the body-replication task). Crucially, these correlations only emerged for the traits that, in the Dualist eyes, ought to be embodied—for non-epistemic traits, and only in the “replication” task (which evaluates embodiment).

This selective association between the transformative sense of Mary’s conscious experience and embodiment intuitions is only expected, given that in the psychological account, it is embodiment intuitions that promote the transformative sense. These results cannot demonstrate that intuitive Dualism *causes* consciousness intuitions. Still, it is clear that the results of Experiment 4 present a formidable challenge to the ontological account, but they are fully in line with the psychological explanation.

## DOES CONSCIOUSNESS REQUIRE A HUMAN BODY?: EXPERIMENT 5

Experiments 1–4 show that, in laypeople’s view, subjective experiences are not all uniformly transformative. Rather, perceptual experiences seem both transformative, they are more plainly anchored in the body, gaining perceptual experience causes a bodily change, and the magnitude of the bodily change and the transformation correlate. These findings are all in line with psychological Essentialism.

As noted, transformative experiences might be seen as such because they seem to more readily tap into a person’s essence. And since the essence is seen as lying deep within the body, conscious perceptual experiences—ones that are readily linked to the body—are seen as more transformative.

Essentialism, however, requires a transformative experience to meet two conditions. Embodiment is the first; the second condition concerns the *kind* of body. Indeed, by default, people attribute essence to living things, but not to inanimate entities—so do young children, for instance (Gelman & Wellman, 1991; Keil, 1986). So, if the transformative force of consciousness arises from essentialism, then to acquire experience, agents not only need *a* body—they need the “right” one (Knobe, 2011). Consciousness, then, ought to be ascribed to living things more than to a silicon AI.

Indeed, past research has found that the ascription of consciousness to robots seems uncanny (Gray & Wegner, 2012). While participants readily ascribe experience to a variety of living agents—to adults, infants, fetuses, and nonhuman animals (Gray et al., 2007), they do not project consciousness to inanimate objects (e.g., vehicles; Arico et al., 2011) and robots (Gray et al., 2007). Specifically, robots seem able to smell and feel pain, anger (Sytsma & Machery, 2010) and happiness (Huebner, 2010).

The reluctance to credit robots with emotive and perceptual experience is in line with the present hypothesis that (a) perceptual and experiences are ascribed to one’s essence (Berent et al., 2020, 2021); and (b) the essence defines living things. Since robots are devoid of essence, they cannot have subjective experiences, as the results indeed suggest.

This conclusion, however, is challenged by methodological limitations. First, in previous studies (Arico et al., 2011; Gray et al., 2007; Huebner, 2010), conscious states were depicted by brief statements (e.g., “capacity to feel pain”). These descriptions may suffice to invoke the familiar experience of people, but not to depict what they might feel like in robots. Accordingly, it is possible that the reluctance to ascribe conscious perceptions and emotions to robots may not be principled, but rather arise from this methodological limitation.

Second, in the absence of “the right body”, robots ought to be devoid not only of subjective perceptual experiences but also of conscious epistemic states. But whether robots are ascribed such experiences is unclear. Huebner (2010) found that people do ascribe robots beliefs (Huebner, 2010). In these experiments, however, the queries were framed using anthropomorphic mentalistic languages (e.g., “believes”). It is thus possible that the positive ascription of subjective beliefs arose because of anthropomorphism.

Accordingly, Experiment 5 further evaluated the ascription of consciousness to humans and robots. In this experiment, participants were presented with detailed descriptions of the subjective (conscious) experiences in two kinds of agents—human and silicon AI. Each kind of agent, in turn, was described as having two types of experiences—a conscious perceptual experience of color vision vs. the conscious knowledge of physics. The human agents were identical to the ones in Experiment 1—Mary, the neuroscience expert, and Jack, the billiards champion. The AI robotic experts were each matched with similar experiences, but the terms “seeing”, and “knowledge” were rephrased, to avoid anthropomorphism. The intuitive psychological account predicts that a conscious perceptual experience ought to seem more transformative only in human agents, but not the AI.

### Methods

#### Participants.

Experiment 5 was assigned to 30 participants. One participant was removed as an outlier; data from one other participant was incomplete. Thus, the analysis of Experiment 5 was based on 28 participants.

#### Materials.

Experiment 5 contrasted the cases of Mary and Jack (as in Experiment 1) with two AI agents, matched to the human agent protagonists with respect to their state before and after the experience. Participants rated whether the human/AI has gained something new through this new experience. Agent order (Human vs. AI vignette) was counterbalanced, and State order (Perception vs. Knowledge vignette) was randomized.

### Results and Discussion

**Figure 7.**
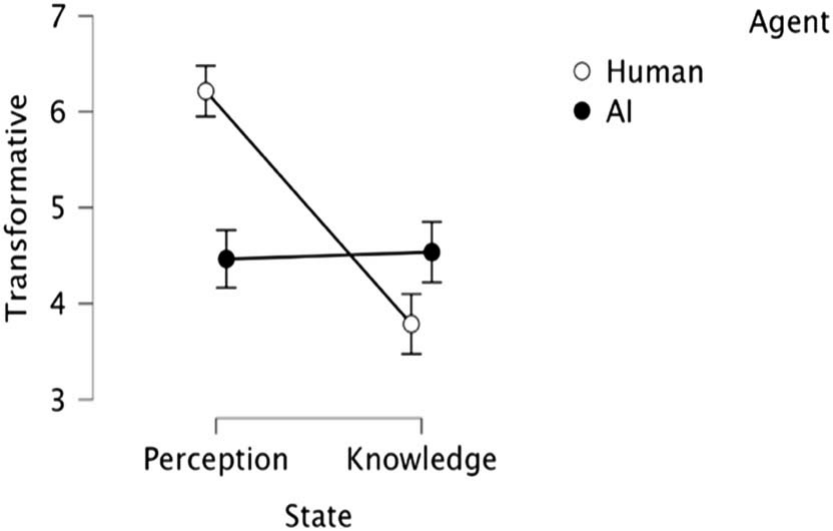
The transformative effect of subjective experience in humans vs. AI. Error bars are normalized *SE*.

Figure 7 plots the mean “transformative” ratings, depending on the mental state in question (Perception vs. Knowledge) and the agent (Human vs. AI). An inspection of the means suggests that, as in Experiment 1, Mary’s conscious perceptual experience seemed more transformative than Jack’s conscious knowledge. This, however, was not the case with the AI.

In line with this impression, the 2 State (Perception vs. Knowledge) × 2 Agent (Human vs. AI) ANOVA yielded a reliable effect of State (*F*(1, 27) = 10.04, *p* < .005, *η*_*p*_^2^ = 0.271) and Agent (*F*(1, 27) = 4.15, *p* = .05, *η*_*p*_^2^ = 0.133), as the perceptual experience was considered more transformative than conscious knowledge, and experiences were seen as more transformative in humans than in AI. Critically, the State × Agent interaction was significant (*F*(1, 27) = 22.34, *p* < .001, *η*_*p*_^2^ = 0.453).

The significant simple main effect of State for the Human agents (*F*(1, 27) = 30.78, *p* < .001) showed that Mary’s conscious perceptual experience was considered more transformative than Jack’s conscious knowledge of physics. This, however, was not the case in AI agents (*F* < 1). Tukey HSD tests further showed that the human perceptual experience was rated as significantly more transformative than all other states (*p* < .01), which, in turn, did not differ (*p* > .9).

Moreover, when compared against the “neutral” midpoint of the rating scale, the human conscious perceptual experience was considered transformative (*t*(27) = 6.54, *p* < .001, *d* = 1.24), whereas conscious knowledge was not (*t* < 1). For the AI, by contrast, neither experience was considered transformative (*t* < 1).

Participants’ reluctance to attribute subjective perceptions to robots converges with the past findings (Arico et al., 2011; Gray et al., 2007; Huebner, 2010; Sytsma & Machery, 2010). Unlike past research (Huebner, 2010), however, participants in Experiment 5 were also reluctant to ascribe to robots epistemic states, possibly, because anthropomorphism was minimized.

Thus, the transformative value of a experience is non-uniform not only across different mental states (in humans) but also across different agents. In human agents, conscious perception seems transformative (relative to knowledge), but this is not the case for AI. This is precisely what Essentialism and Dualism predict.

## GENERAL DISCUSSION

Why is consciousness such a hard problem? Do our troubles with consciousness ultimately arise from ontology—from the fact that consciousness *is* distinct from the physical, or from our psychological bias to *view* it as such?

To address this question, here, I presented an in-depth analysis of one of Chalmers’ “problem intuitions”—that of Mary in the black and white room (as noted, all cases are discussed in the SM). When Mary—the vision neuroscientist—sees color for the first time, her experience seems to us utterly new, perhaps transformative.

The ontological account asserts that this is so because consciousness is irreducible to the physical. The psychological account agrees that the transformation arises, in part, from a tension between the physical and the non-physical. But in the psychological account, this tension is likely an illusion. It reflects not how things *are* (ontologically) but how they *seem* to us. The illusion arises from two psychological constraints—Dualism and Essentialism.

To determine whether an experience is transformative, we evaluate whether it sheds light on a person’s essence, which lies deep within their body. Experiences that we readily anchor in the body thus ought to seem more likely to pertain to one’s essence, hence, more transformative. Color vision is a case in point—vision appears to arise from the eyes. Accordingly, Mary’s visual experience with colors ought to seem particularly transformative.

People, however, are also Dualist (Bloom, 2004), so they consider some mental states as ethereal, and epistemic states seem particularly ethereal (e.g., Berent, 2023). Mary’s knowledge (prior to seeing color) thus ought to seem disembodied, hence, irrelevant to her essence, and, consequently, unremarkable (i.e., not transformative).

This psychological account stands in sharp contrast to the ontological explanation (as outlined in Chalmers, 1996, and extended by the psychological linking hypotheses in (1)). In the ontological account, transformative experiences are disembodied; in the psychological account, they are embodied. Furthermore, the ontological account ought to predict that that all experiences are uniformly transformative, and they need not be confined to human agents alone. The psychological account, by contrast, predicts that only conscious perceptions should be transformative (not conscious knowledge), and this should be the case in humans, but not in robots. Experiments 1–5 tested these predictions.

Experiments 1–2 show that the transformative force of experience isn’t uniform: subjective perceptual experiences are more transformative than knowledge. Moreover, the transformative potential of experience is linked to its embodiment. Gaining perceptual experience seems both transformative and embodied, whereas gaining conscious knowledge seems neither. Furthermore, intuitions about transformation and embodiment correlate.

Experiments 3–4 show that, in laypeople’s view, gaining experience causes changes in one’s body: as one becomes consciously aware of color, that experience is more likely to “show up” in the brain (relative to a strictly matched subliminal state involving the same sensation). Experiment 4 further shows that participants indeed lean toward intuitive Dualism, and that their intuitions about the embodiment of psychological traits (generally) selectively predict their transformative intuitions concerning Mary’s experience. Finally, Experiment 5 suggests that only humans seem privy to such transformation, but not robotic AI.

Taken as a whole, the results of Experiments 1–5 support Chalmers’ assertion that Mary’s case indeed seems novel and significant; in fact, it’s considered “transformative”. Nonetheless, these results challenge the ontological explanation for why the transformation arises.

A reviewer of this paper, however, was concerned that the subjective perceptual experiences might seem more transformative because the magnitude of the change they elicit (i.e., the difference between the perceptual experience relative to the previous state) is more dramatic or more emotive relative to the magnitude of the change engendered by conscious knowledge, and these differences in “magnitude” could confound the results.

I believe this is unlikely. To present a confound, one would need to show how differences in “magnitude” ***cause*** the present findings (e.g., how the greater magnitude of the perceptual change causes people to consider perceptual experiences as more embodied). No such explanation is offered. Moreover, Experiments 3–4 make it clear that perceptual experiences seem transformative and embodied even when they are *not* compared against knowledge (i.e., when differences in “magnitude” do not exist). So, not only does the “magnitude” concern fail to explain the findings of Experiments 1–2, but it certainly cannot capture the findings as whole.

A far more likely explanation, then, is that, if “magnitude” differences exist (i.e., if perceptual changes seem more dramatic), then these differences are the ***consequence*** of gaining experiences (i.e., the perceptual change seems more dramatic because perception seems more likely to give rise to a transformative experience), rather than their cause (i.e., perception gives rise to transformative experience because such experiences are somehow more dramatic/emotive). This possibility can be readily captured by the psychological explanation advanced here. Indeed, it is precisely because people consider conscious perceptions more transformative (due to intuitive Dualism and Essentialism) that such experiences seem transformative, embodied, and perhaps also more dramatic. Crucially, if “magnitude” differences are the consequence of gaining consciousness, then they cannot possibly confound the present investigation of its causes.

Still, several limitations of these findings are noteworthy. First, the results cannot ascertain the source of consciousness intuitions. While the present findings are certainly in line with the Essentialist explanation, they do not specifically demonstrate that these intuitions arise from Essentialism, nor do they show how Essentialism promotes the sense of transformation. Likewise, while the results of Experiments 4 provide several indications that performance is governed by intuitive Dualism, they fall short of providing evidence for the causal role of Dualism. Still, the intuition that some mental states seem more embodied than others has no basis in science, nor is it predicted by the ontological explanation. So, clearly, the bias is psychological.

Second, these results cannot ascertain the scope of the bias, as these results are only informed by a specific contrast—between the experiences of perception vs. knowledge, and, furthermore, the perceptual experience specifically concerns the case of Mary in the black and white room. Another limitation arises from the fact that participants are all members of large-scale societies (see Table S2). Given that the ontological account was prominently supported by Mary’s case and by the intuitions of Western readers, the present results may be of nonetheless of interest.

Third, I note that the experimental evaluation of the ontological account is premised on a number of psychological linking hypotheses that go beyond Chalmers’ (1996) original claims. Accordingly, the shortcomings of the ontological account (as outlined in (1)) could arise from the inadequacy of the linking hypotheses, rather than Chalmers’ own analysis.

What consciousness really is, and whether it is indeed irreducible to the physical, is not a question that this research can settle. But these results make it clear that our intuitive view of consciousness is systematically biased. Thus, the “hard problem of consciousness” arises, in part, from human psychology.

## ACKNOWLEDGMENTS

I thank David Chalmers, for discussion of this research, and Dana Scott, for sparking my interest in “the hard problem”. I also thank Jorge Morales and my lab members, especially, Alexzander Sansiveri and Kumud Joshi, for helpful comments, and Eliza Rice, for editorial assistance. This research was not supported by any funding source.

## DATA AVAILABILITY STATEMENT

All data are appended as a supplementary material file.
