# Supplementary material for: The “Hard Problem of Consciousness” Arises from Human Psychology
Source: Open Mind (Camb). 2023 Aug 11;7:564–87. doi: 10.1162/opmi_a_00094 (PMC10449398; doi:10.1162/opmi_a_00094)
Supplement: Supplementary file 1 [file opmi-07-564-s001.docx]

**The “hard problem of consciousness” arises from human psychology**

***Supplementary Materials***

Iris Berent

Northeastern University

*Corresponding author: Iris Berent

[i.berent@northeastern.edu](mailto:i.berent@northeastern.edu)

1. ***ADDITIONAL “PROBLE INTUITIONS”***

Chalmers (2009) demonstrates the “hard problem” using numerous cases that lead readers to intuit that indeed, consciousness is distinct from the physical—the so-called “problem intuitions”. In the main text, I focus on one of these cases. Here, I briefly describe the remaining problems; the next section outlines how these intuitions may be captured by the psychological account.

Chalmers presents two lines of arguments that consciousness is irreducible to the physical. The first shows that consciousness can dissociate from physical properties (see (1)).

One such case is presented by zombies—creatures that are identical to us physically, yet devoid of consciousness. Chalmers claims that (1) if such zombies are identical to us, then, necessarily, they are identical to us (since, if x is identical to y, then, necessarily, x is identical to y; Kripke, 1980); (2) zombies are conceivable; and (3) if zombies are conceivable, they are possible. Chalmers thus concludes that our experiences are not identical to our brain states[[1]](#footnote-1).

The “color spectrum” likewise elicits “problem intuition”. Here, we are invited to conceive of another human being that is identical to us physically, but not psychologically—they do not possess our subjective experiences. When presented with the portion of the color spectrum that we perceive as red, that person would subjectively experience blue. And if the same physical conditions can give rise to different experiences, then consciousness does not logically supervene on the physical. Both arguments (the zombies and “color spectrum”), then, show consciousness is distinct from physical facts.

1. A summary of the problem intuitions.
   1. Consciousness can dissociate from physical properties.
      1. zombies.
      2. Color spectrum.
   2. Consciousness is irreducible to knowledge of physical facts.
      1. Mary in the black and white room.
      2. Silicon AI.
      3. Consciousness vs. functional analysis.

Chalmers’ second argument shows that consciousness is irreducible to *knowledge* of physical facts. Mary in the black and white room (discussed in the main text) presents one such example; a silicon AI presents another. Chalmers notes that we can readily think of a silicon AI that possesses full knowledge of color vision but is devoid of consciousness. Finally, it is possible to functionally analyze how a conscious experience causes other mental states to emerge, but doing so still fails to define what consciousness is. Chalmers (Chalmers, 1996) considers these cases as evidence that consciousness is not logically supervenient on the physical. And if so, physicalism is false.

***The view from intuitive psychology***

1. *Consciousness vs. the external physical world*

Chalmers’ first line of evidence contrasts conscious agents with mindless entities—either zombies or physical phenomena (e.g., color spectra). To explain why consciousness is inexplicable by the physical properties, one does not necessarily need to assume that the mind, consciousness included, is a special ontological substance or property. Suffice it to assume we *believe* it is.

As noted, people are intuitive Dualism (for review, see the main text). Now, given that consciousness is a mental state, and mental states, generally, seem ethereal, it is only expected that people will consider consciousness ethereal, so they will struggle to anchor it in the physical. If so, copying one’s body won’t copy one’s consciousness. Likewise, a zombie that preserves one’s body is not guaranteed to preserve either one’s psyche, generally, or one’s consciousness, specifically (FigureS1A).

A.

B.

FigureS1. Dualism dissociates consciousness from the physical. This gives rise to mindless zombies (A) and dissociations between perception and the color spectrum (B).

Since the Dualist struggles to conceive of the physical as the cause of the mental (Berent, 2021a), the physical and the metnal seem to readily dissociate: a single color spectrum can conceivably lead you and me to distinct conscious experiences, and conversely, our shared conscious experience could arise from entirely distinct color spectra (see Figure S1B). Accordingly, conscious experience is unpredictable from the physical. This is how intuitive Dualism explains why consciousness is “hard”.

1. *Consciousness vs. knowledge*

Consciousness, however, might seem distinct from the external physical world (as shown in the previous “problem intuitions”) but also from knowledge—another mental state. And indeed, we feel that, upon Mary’s first encounter with color, her conscious experience offers her something new and transformative—above and beyond her knowledge of the physical.

This intuitive explanation for this case is detailed in the Main Text. Here, I wish to explain why it’s the consciousness vs. knowledge cases (for the zombie and color spectrum), the conscious experience seems ethereal, wheatears in Mary’s case, it seems embodied. The difference, I suggest, arises because the former cases primarily invoke Dualism, whereas the latter also underscores Essentialism. We now consider how this difference arises from the framings of these problems.

Two factors contribute to this difference. First, in the former cases (zombies, color spectrum), consciousness is contrasted with the physical (with a person’s body, and with the color spectrum, respectively), so by comparison, consciousness seems ethereal. Mary’s case, by contrast, compares two psychological states to each other—perception and knowledge. And against the backdrop of ethereal knowledge, subjective perception would now seem *relatively* embodied—more so than knowledge.

Second, the first set of problems (zombie and the color spectrum), we compare two agents to each other—me vs. my zombie, and the perceiver with one color experience and another perceiver. Each such agent is invariant (it incurs no changes)—the problem invites us to contrast *between* the agents. And in each case, we determine whether conscious (a mental state) can dissociate from the physical. For the Dualist, the answer is yes, and by comparison to the physical (the body, and color spectrum), consciousness further seems ethereal.

Mary, by contrast, undergoes change, and the problem implicitly invites us to determine evaluate its significance. To evaluate changes to living things, generally (Keil, 1986) and to their self, specifically (Berent & Platt, 2021; De Freitas & Cikara, 2018; De Freitas, Cikara, Grossmann, & Schlegel, 2017; De Freitas et al., 2018; De Freitas, Tobia, Newman, & Knobe, 2017; Strohminger, Knobe, & Newman, 2017), we invoke Essentialism. And as noted, we align the essence with the body (for review: Berent, 2021b). Accordingly, in Mary’s case, we focus on whether her experience has tapped into her embodied essence, and perceptions fit the bill (more so than knowledge). So, in Mary’s case, her conscious experience seems relatively embodied (Figure 2A).


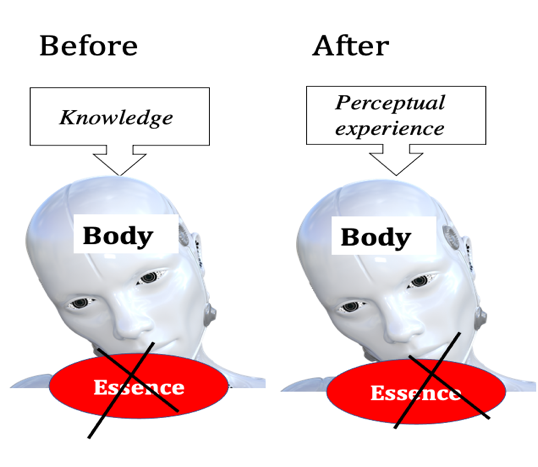


B.

A AA

Figure 2. Knowledge vs. conscious experience of color vision in humans vs. silicon AI.

The next problem—that of the silicon AI—likewise invites intuitions about the agents’ essence, because at stake is whether the agent is inherently endowed with the capacity to experience conscious states—a question of essence (Figure 2B). But the AI body is comprised of silicon—an inorganic substance, whereas Mary’s example suggests that her essence is partly a property of flesh. Since AI lacks the “right” stuff of essence, the AI body cannot support consciousness, in line with past research (Gray, Gray, & Wegner, 2007; Huebner, 2009; Sytsma & Machery, 2010). For the same reason, its experience of color vision is hardly transformative—it does not tap into its core or reveal anything about its hidden essence (as Experiment 5 indeed suggests).

Chalmers’ final argument—that consciousness cannot be functionally defined—could likewise be the legacy of Essentialism. Indeed, essentialist reasoning is asymmetric: the essence of a living kind seems to cause its surface traits, but it is uncaused by them (Keil, 1986), and children also weigh causes more heavily than effects in drawing an inference about kinds (Ahn et al., 2001). If we essentialize consciousness (more than knowledge), then consciousness ought to appear as the “uncaused causer”: it can be linked to other mental states, but it cannot be defined by them.

Summarizing, then, the analysis advanced here demonstrates how intuitive psychology (specifically, the interaction between Dualism and Essentialism) might be able to capture each of Chalmers’ illustrations. I suggest that the two sets of problems (contrasting consciousness with the external world vs. knowledge) elicit different intuitions because, in the former (consciousness vs. the external world), the problem framing underscores Dualism whereas in the latter set (consciousness vs. knowledge), Essentialism comes into play, as the problem either invokes a change to the agent (in Mary’s case) or underscores the agents’ inherent potential (in the AI’s case and in the analysis of functional states).

Whether these proposals indeed capture laypeople’s intuitions is a question for future research. Here, I demonstrate that the theory advanced in the main text can potentially capture each of these problems.

**2. *METHODS***

**Experiment 1**

*Participants.* Thirty participants took part in this experiment. They were adult, native English speakers, who self-identified as having no neurological or language disorders, and they also had no known diagnosis of autism. In this and all subsequent experiments, participants were recruited from Prolific. Sample size was based on pilot research, suggesting that the selected sample size was sufficient to attain a power of 80% at the alpha level of .05.

Prior to analysis, we inspected the results for outliers (responses falling 2.5SD beyond the cell mean); this procedure was applied to this and all subsequent experiments. Two such participants were excluded. Three additional participants did not finish the experiments. Participants’ characteristics and area of residence are presented in Table S1-S2.

**Table S1. Demographics of participants in Exp.’s 1-4.**

| ***Exp.*** | ***N*** | ***Gender*** | | ***Age*** | | ***Ethnicity*** | | |
| --- | --- | --- | --- | --- | --- | --- | --- | --- |
|  |  | *Female* | *Male* | *Mean* | *SD* | *White* | *Black* | *Other* |
| 1 | 25 | 12 | 13 | 37.84 | 11.6 | 20 | 4 | 1 |
| 2 | 57 | 27 | 30 | 33.85 | 11.54 | 46 | 7 | 4 |
| 3 | 30 | 15 | 15 | 34.16 | 15.54 | 16 | 10 | 1 |
| 4 | 56 | 28 | 28 | 36.93 | 13.09 | 46 | 4 | 6 |
| 5 | 28 | 20 | 8 | 36.28 | 12.95 | 21 | 5 | 2 |

**Table S2. Area of residence of participants in Exp.’s 1-4.**

| ***Exp.*** | ***Area of residence*** | | | | | |
| --- | --- | --- | --- | --- | --- | --- |
|  | *North America* | *UK/Ireland* | *Europe* | *Africa* | *Australia/New-Zealand* | *South America* |
| 1 | 0 | 17 | 0 | 6 | 2 | 0 |
| 2 | 9 | 35 | 4 | 5 | 3 | 1 |
| 3 | 1 | 16 | 2 | 9 | 1 | 1 |
| 4 | 10 | 37 | 1 | 4 | 4 | 0 |
| 5 | 19 | 4 | 3 | 0 | 2 | 0 |

*Materials.* Participants were presented with two vignettes, featuring two protagonists—Mary and Jack. Each such protagonist was said to have undergone a change, leading to the acquisition of a new conscious experience. Prior to this experience, Mary had an explicit understanding of color vision; her new experience consisted of the conscious experience of color vision (seeing a red rose). Jack, by contrast, had a tacit understanding of how balls move, as he was an expert billiards player; his new experience was the acquisition of the conscious knowledge of the physical laws of motion, via a crash course in physics.

For each protagonist, participants were asked to answer three questions. The first was to rate how transformative was the new conscious experience: whether, as a result of this experience, the protagonist has gained something new (1=not at all; 7=very greatly).

Two additional questions invited participants to evaluate whether the protagonist’s states—before and after the conscious experience—are likely to show up in their brain. To evaluate the “before” state, participants were asked to compare the protagonist before the acquisition of the conscious experience to their twin sister/brother who lacked that experience. Their task was to indicate whether the protagonist’s brain would look differently from their twin on an fMRI scan. To evaluate the embodiment of the new conscious experience, participants were next asked to compare the brain scan of the protagonist before and after the conscious experience. In both questions, participants judged whether the scans differed (1=very similar; 7=very different). The order of the two vignettes was randomized. The materials for this and all subsequent experiments are presented in Appendix I.

**Experiment 2**

*Participants.* Two groups of participants (N=30 each) were assigned to Experiments 2. Of the 60 assigned participants, two did not complete the task, and one was removed as an outlier. Sample size was doubled arbitrarily, as in this experiment, the effect of State was manipulated between participants.

*Materials.* Each group of participants was assigned to one vignette. One vignette featured the case of Mary in the black and white room, who had explicit knowledge of color vision, and subsequently, acquired a conscious visual experience by seeing a red rose for the first time. The second vignette featured Susan, a renowned artist who specializes in painting red roses. Susan has an intuitive “feel” of how color works, and she has subsequently acquired conscious knowledge of the principles of color chemistry, optics and neuroscience. Participants evaluated the transformative value of the conscious experience and the likelihood of each protagonist’s mental state (before vs. after gaining consciousness) showing up in the brain (as in Experiment 1), using a 1-7 scale (as in Experiment 1).

**Experiment 3**

*Participants.* 30 participants were assigned to Experiment 3. One outlier was removed from the analysis.

*Materials.* Participants read a single vignette featuring Mary in the black-and-white room. Next, they were invited to suppose Mary underwent an fMRI scan that examined her brain response to her first encounter with a red rose—either subliminally and unconsciously, or consciously. Participants evaluated (on a 1-7 scale) how likely each experience is to “show up” in her brain, and how transformative is each experience.

**Experiment 4**

*Participants.* 60 participants were assigned to Experiment 4. Five outliers were removed from the analysis.

*Materials.* The experiment featured two set of tasks. The first set probes intuitions concerning Mary’s case, exactly as in Experiment 3. The second set of task assessed Dualism using the body-replication and afterlife tasks. The order of the two tasks (replication/afterlife) was counterbalanced, and each task featured half of the traits (balanced for type—epistemic vs. non-epistemic) and counterbalanced across the two tasks.

**Experiment 5**

*Participants.* Another group of 30 participants took part in Experiment 4. One participant did not complete the experiment; another was removed as an outlier.

*Materials.* Participants were presented with four vignettes, featuring four protagonists—two humans and two AI agents. Each such agent underwent a change, leading to the acquisition of an explicit experience with a mental state that was previously tacit. Two of those states involved a change from the prior knowledge of vision to a visual experience with the color red. In the other two cases, the agent had a tacit understanding of ball-motion, as evident by their expert command of billiards, and they subsequently acquired an explicit understanding of the physical laws of motion. For the human agent, the new explicit state was labeled as “conscious” and the previous states were labeled “knowledge” and “gut intuition”, respectively. To avoid orthomorphism, for the AI, the new state was labeled as either an “encounter” (with a red rose) or explicit programming of the laws of physics into the AI. Participants were asked to rate whether the human/AI has gained something new by this new (conscious/explicit) experience using a 1-7 scale (1=not at all, 7=very greatly). The order of the two agents (Human vs. AI) was counterbalanced, and the order of States (Perception vs. Knowledge) was randomized.

**References**

Ahn, W.-K., Kalish, C., Gelman, S. A., Medin, D. L., Luhmann, C., Atran, S., . . . Shafto, P. (2001). Why essences are essential in the psychology of concepts. *Cognition, 82*(1), 59-69. doi: 10.1016/S0010-0277(01)00145-7

Berent, I. (2021a). Can the mind command the body? *Cognitive science, 45*(2), e13067.

Berent, I. (2021b). On the matter of essence. *Cognition, 213*, 104701.

Berent, I., & Platt, M. (2021). The true “me”—mind or body? *Journal of Experimental Social Psychology, 93*, 104100.

Chalmers, D. J. (1996). *The conscious mind: in search of a fundamental theory*. New York: Oxford University Press

Chalmers, D. J. (2009). The Two‐Dimensional Argument Against Materialism. In A. Beckermann, B. P. McLaughlin & S. Walter (Eds.), *The Oxford Handbook of Philosophy of Mind* (pp. 0): Oxford University Press.doi: 10.1093/oxfordhb/9780199262618.003.0019

De Freitas, J., & Cikara, M. (2018). Deep down my enemy is good: Thinking about the true self reduces intergroup bias. *Journal of Experimental Social Psychology, 74*, 307-316. doi: 10.1016/j.jesp.2017.10.006

De Freitas, J., Cikara, M., Grossmann, I., & Schlegel, R. (2017). Origins of the belief in good true selves. *Trends in Cognitive Sciences, 21*(9), 634-636. doi: 10.1016/j.tics.2017.05.009

De Freitas, J., Sarkissian, H., Newman, G. E., Grossmann, I., De Brigard, F., Luco, A., & Knobe, J. (2018). Consistent Belief in a Good True Self in Misanthropes and Three Interdependent Cultures. *Cognitive science, 42 Suppl 1*, 134-160. doi: 10.1111/cogs.12505

De Freitas, J., Tobia, K. P., Newman, G. E., & Knobe, J. (2017). Normative judgments and individual essence. *Cognitive science, 41*(Suppl 3), 382-402. doi: 10.1111/cogs.12364

Gray, H. M., Gray, K., & Wegner, D. M. (2007). Dimensions of Mind Perception. *Science (American Association for the Advancement of Science), 315*(5812), 619-619. doi: 10.1126/science.1134475

Huebner, B. (2009). Commonsense Concepts of Phenomenal Consciousness: Does Anyone Care About Functional Zombies? *Phenomenology and the Cognitive Sciences, 9*, 133-155. doi: 10.1007/s11097-009-9126-6

Keil, F. C. (1986). The acquisition of natural kind and artifact term. In W. Demopoulos & A. Marras (Eds.), *Language Learning and Concept Acquisition* (pp. 133-153). New Jersey: Ablex: Norwood,

Kripke, S. A. (1980). *Naming and necessity*. Cambridge, Mass.: Harvard University Press

Strohminger, N., Knobe, J., & Newman, G. (2017). The True Self: A Psychological Concept Distinct From the Self. *Perspectives on Psychological Science, 12*(4), 551-560. doi: 10.1177/1745691616689495

Sytsma, J., & Machery, E. (2010). Two conceptions of subjective experience. *Philosophical studies, 151*(2), 299-327. doi: 10.1007/s11098-009-9439-x

***3. APPENDICES***

**Appendix I: Experimental materials**

**Materials in Experiment 1**

Mary is a neuroscientist—she is one of the world’s leading experts on color vision. She knows exactly how color vision happens in the brain, and why we see color. For example, she understands what area of the brain encodes the color “red” (as distinct from “blue”), and how the neural firing gives rise to this specific percept.  Mary fully understands how the brain gives rise to color vision.

Mary, however, has never seen color herself. She was brought up in a black-and-white room. Although she has seen many shades of black, gray, and white, Mary has never seen any other color. So, Mary knows everything there is to know about how people see “red.” In particular, she understands how the brain interprets certain wavelengths as “red” and what physical and neural processes make us see “red.” But she has never experienced how “red” feels.

Now, suppose Mary leaves her black-and-white room for the first time and sees a red rose. She watches the rose intensely, and consciously registers its brightness and shine. Has she gained something new?

How transformative is Mary’s experience seeing the color red? How much has her grasp of “red” changed by seeing the red rose?


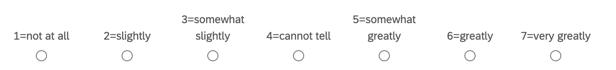


Did this new experience of color register in her brain? How does this experience with color differ from her previous understanding of visual neuroscience?

To be more concrete, consider Mary while she is still in the black-and-white room (before seeing the red rose). Suppose we were to scan Mary’s brain using an fMRI machine and compare it to a scan of Jane--Mary’s identical twin sister, who also lives in a black-and-white room, but knows nothing about neuroscience (nor has she ever seen “red”). Would the scan of Mary, the expert on the neuroscience of color-vision, look different than the scan of Jane, who knows nothing about color vision?


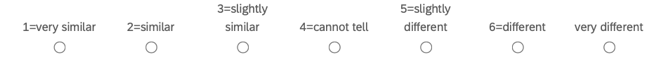


Now, consider Mary’s conscious experience of color vision after she has seen a rose for the first time. Would that experience elicit a detectable change in her brain?

 For concreteness, suppose we scanned Mary’s brain immediately after she had seen a red rose for the first time, and compared that scan to the scan obtained while Mary was in the black-and-white room (before seeing the red rose). Is Mary’s experience with color detectable in her brain? That is, would the two scans (before/after seeing the red rose) differ?


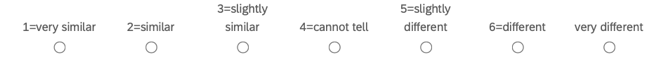
Jack is a professional billiards player—he is considered the world’s best player ever. Jack knows exactly how to launch a ball so it hits its target at the right angle and velocity. In the past ten years, he has won practically every game played. So clearly, Jack has an intimate and precise intuitive grasp of how billiard balls move.

Jack, however, has no explicit understanding of the laws of physics. He has never finished high school. Although Jack constantly interacts with balls, he did not take a single physics course in his life. So, Jack has perfect “gut” intuitions of how balls move. In particular, he can superbly predict their velocity and trajectory, and how their speed varies depending on the friction with the table. But Jack has never heard the term “momentum” or Newtonian physics.

Now, suppose that, on the advice of his agent, Jack takes a crash course in physics, where he learns the laws of motion, as they applied to launching billiard balls. Jack can now describe these laws perfectly, and he is consciously aware of them. Has he gained something new?

 How transformative is Jack’s crash course in physics? How much has his grasp of billiards changed by learning the laws of physics?


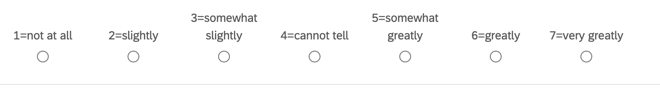

 Did this new knowledge of physics register in his brain? How does his conscious understanding of physics differ from his previous intuitive grasp?

To be more concrete, consider Jack before his crash course on physics. Suppose we were to scan Jacks brain using an fMRI machine and compare it to a scan of John--Jack’s identical twin brother, who knows nothing about billiards (or physics). Would the scan of Jack, the billiards expert, look different than the scan of John, who knows nothing about billiards?


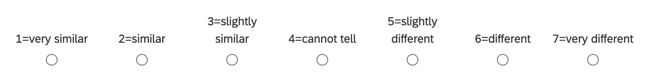


Now, consider Jack’s conscious knowledge of the laws of physics after taking the crash course. Would that experience elicit a detectable change in his brain?

For concreteness, suppose we scanned Jack’s brain immediately after he took the physics course, and compared that scan to the scan obtained before the physics course (before being becoming aware of the laws of physics). Is Jack’s knowledge of the laws of physics detectable in his brain? That is, would the two scans (before/after the physics course) differ?
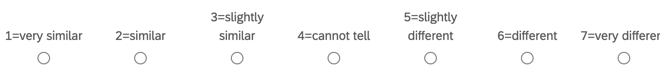


**Materials in Experient 2**

Mary is a neuroscientist—she is one of the world’s leading experts on color vision. She knows exactly how color vision happens in the brain, and why we see color. For example, she understands what area of the brain encodes the color “red” (as distinct from “blue”), and how the neural firing gives rise to this specific percept.  Mary fully understands how the brain gives rise to color vision.


Mary, however, has never seen color herself. She was brought up in a black-and-white room. Although she has seen many shades of black, gray, and white, Mary has never seen any other color. So, Mary knows everything there is to know about how people see “red.” In particular, she understands how the brain interprets certain wavelengths as “red” and what physical and neural processes make us see “red.” But she has never experienced how “red” feels.

Now, suppose Mary leaves her black-and-white room for the first time and sees a red rose. She watches the rose intensely, and consciously registers its brightness and shine. Has she gained something new?

How transformative is Mary’s experience seeing the color red? How much has her grasp of “red” changed by seeing the red rose?


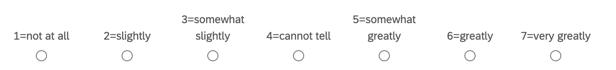


Did this new experience of *seeing* color register in her brain? How does this conscious experience of seeing color differ from her previous *knowledge* of visual neuroscience?

To be more concrete, consider Mary while she is still in the black-and-white room (before seeing the red rose). Suppose we were to scan Mary’s brain using an fMRI machine and compare it to a scan of Jane--Mary’s identical twin sister, who also lives in a black-and-white room, but knows nothing about neuroscience (nor has she ever seen “red”). Would the scan of Mary, the renowned expert on the neuroscience of color-vision, look different than the scan of Jane, who knows nothing about neuroscience?


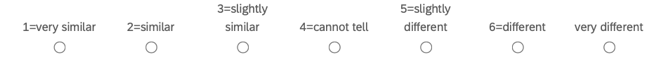


Now, consider Mary’s conscious experience of color vision after she has seen a rose for the first time. Would that experience elicit a detectable change in her brain?

 For concreteness, suppose we scanned Mary’s brain immediately after she had seen a red rose for the first time, and compared that scan to the scan obtained while Mary was in the black-and-white room (while she only had scientific knowledge of color vision, but hasn’t yet seen “red” herself).Is Mary’s conscious experience with *seeing* color detectable in her brain? That is, would the two scans (before/after seeing the red rose) differ?


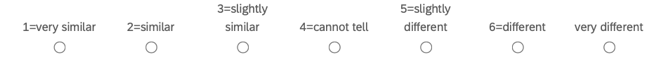


Susan is a renowned artist—her work has been featured in many museums worldwide. Susan is especially famous for her large images of red roses, featuring lush, vivid shades of red, which she achieves thanks to special pigments that Susan concocts herself. The result is stunning. When people look at those images, they are mesmerized by the lavish, luxurious hues. Susan, no doubt, knows how to make color “speak”.

Susan, however, has no explicit understanding of color chemistry or psychology. She has never finished high school. Although Susan masterfully uses color in her paintings, she has not taken a single course in science or the psychology of vision. So, Susan has perfect “gut” intuition about how to mix chemical substances to create the perfect hue and how to manipulate color so it strikes the human viewers. But Susan has never heard of terms like “photons” “macular pigment”, or “photoreceptors”.

Now, suppose that, on the advice of her agent, Susan takes a crash course in the science of color vision, where she learns about the principles of color chemistry, optics and the psychology of color vision. Susan can now describe these principles perfectly, and she is consciously aware of how the science of color works—why the substances she concocts yield theses special shades of red, and why viewers perceive them in the way they do. Has she gained something new?

How transformative is Susan’s crash course in the science of color vision? How much has her grasp of painting with color changed by learning these scientific principles?


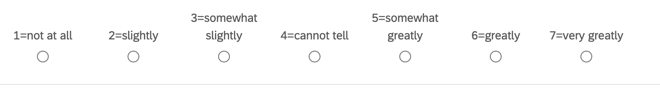

Did this new *knowledge* of the science of color vision register in her brain? How does her conscious scientific *knowledge* of color vision differ from the previous intuitive grasp she had though her painting?

To be more concrete, consider Susan before her crash course on the science of color vision. Suppose we were to scan Susan’s brain using an fMRI machine and compare it to a scan of Diane—Susan’s identical twin sister, who cannot paint (and knows nothing about science of color vision). Would the scan of Susan, with the great artistic “gut” for color, look different than the scan of Diane, who has no such intuitive grasp?


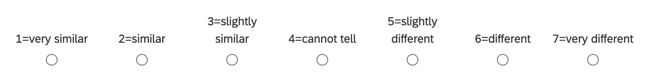


Now, consider Susan’s conscious knowledge of the principles of color vision after taking the crash course. Would that experience elicit a detectable change in her brain?

For concreteness, suppose we scanned Susan’s brain immediately after she took the science course, and compared that scan to the scan obtained before the science course (while she only had the artistic, intuitive “gut” feel for color, but hadn’t yet had any explicit knowledge of

the principles of color vision). Is Susan’s conscious *knowledge* of the principles of color vision detectable in her brain? That is, would the two scans (before/after the science course) differ?

**Materials in Experiment 3**

Mary is a neuroscientist—she is one of the world’s leading experts on color vision. She knows exactly how color vision happens in the brain, and why we see color. For example, she understands what area of the brain encodes the color “red” (as distinct from “blue”), and how the neural firing gives rise to this specific percept.  Mary fully understands how the brain gives rise to color vision.


Mary, however, has never seen color herself. She was brought up in a black-and-white room. Although she has seen many shades of black, gray, and white, Mary has never seen any other color. So, Mary knows everything there is to know about how people see “red.” In particular, she understands how the brain interprets certain wavelengths as “red” and what physical and neural processes make us see “red.” But she has never experienced how “red” feels.

John, a fellow scientist hears about Mary and wonders how would Mary’s brain react if she were to see color for the first time. To evaluate this question, the scientist tests Mary in two experiments.

In the **first** hypothetical experiment, an image of a red rose flashes on the computer screen for just a fraction of a second. John knows this is too quick for people to recognize what they see. So, if John were to ask Mary “what did you see”, she would say “nothing”. Still, past research suggests that the image is perceived, as immediately after seeing the image, the word *rose* readily comes to mind (e.g., people read *rose* more quickly). Now, suppose that, as the subliminal image of the red rose is presented for the first time, Mary’s brain is being scanned.

How likely is it that the experience of subliminally seeing the color “red” for the first time (without Mary’s conscious awareness) would “show up” in Mary’s brain scan?


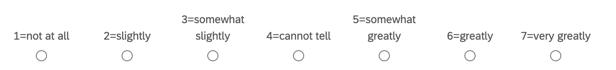


How transformative would Mary’s subliminal experience of unconsciously seeing the color “red” for the first time be? How much would her grasp of “red” change by subliminally seeing the red rose?


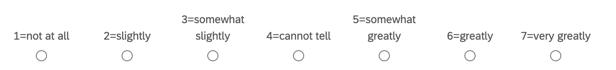


In a **second** hypothetical experiment, Mary sees a red rose on the screen. Now, the image is presented for five seconds, and the rose is clearly visible. So, if John were to ask Mary “what do you see”, Mary says “red rose”. Suppose that, as this clearly visible image of the red rose is presented for the first time, Mary’s brain is being scanned.

How likely is it that the experience of consciously seeing the color “red” for the first time would “show up” in Mary’s brain scan?


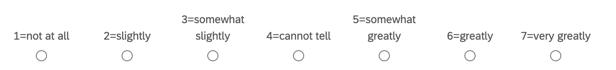


How transformative would Mary’s experience of consciously seeing the color “red” for the first time be? How much would her grasp of “red” change by consciously seeing the red rose?


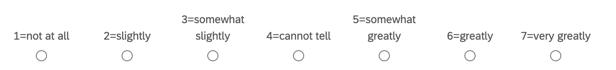


**Materials in Experiment 4 (Dualism tasks)**

1. **Replication task**

In what follows, we are asking you to reason about a hypothetical scenario.

Suppose it were possible to grow a replica of the body of an adult human donor. The replica preserves every aspect of the human body and brain. In particular, suppose that the body of the replica looks and works precisely like a normal human body. Similarly, the brain replica is identical in all respects to the brain of the adult donor.

Below is a list of various traits that define the human donor. Will these traits emerge in its replica?

Please indicate your answer as either 1=yes, this trait will emerge in the replica OR 2=no, this trait will not emerge in the replica.

Do you have any questions?

We will now start with a very brief practice session, if you have any questions, you can ask them at any time during the practice. Then, we will move to the main experimental session.

**ii. Afterlife task**

In this experiment, we examine people’s beliefs about the afterlife. Whether or not there is an afterlife, is of course unknown. But for the purpose of this experiment, we invite you to assume that after people die, they do continue to exist in some capacity. Your task is to reason about which human traits are likely to be maintained in the afterlife.

Below is a list of human traits. Then, please determine the following: if an afterlife exists, will these traits emerge in the afterlife?

Please indicate your answer as either 1=yes, this trait will emerge in the afterlife OR 2=no, this trait will not emerge in the afterlife.  Thank you!

Do you have any questions?

We will now start with a very brief practice session, if you have any questions, you can ask them at any time during the practice. Then, we will move to the main experimental session.

**III. Trait list**

| **ID** | **Trait** | **Type** |
| --- | --- | --- |
| 1 | Anger in response to hostility | Non-epistemic |
| 2 | Love for one's family | Non-epistemic |
| 3 | Contentment with one's life | Non-epistemic |
| 4 | Disgust by feces | Non-epistemic |
| 5 | Excitement towards an opportunity | Non-epistemic |
| 6 | Fear of danger | Non-epistemic |
| 7 | Happiness at the birth of one's child | Non-epistemic |
| 8 | Joy of being | Non-epistemic |
| 9 | Pride in one's accomplishments | Non-epistemic |
| 10 | Sadness from a friend's death | Non-epistemic |
| 11 | Shame from one's shortcomings | Non-epistemic |
| 12 | Surprise at an unexpected event | Non-epistemic |
| 13 | Trust in one's family | Non-epistemic |
| 14 | Jealousy towards a lover | Non-epistemic |
| 15 | Envy at a competitor's success | Non-epistemic |
| 16 | Empathy towards a person in need | Non-epistemic |
| 17 | Admiration for wisdom | Non-epistemic |
| 18 | Pain from witnessing illness and death | Non-epistemic |
| 19 | Hope for a better future | Non-epistemic |
| 20 | Affection towards others | Non-epistemic |
| 21 | Gripping objects by hand | Non-epistemic |
| 22 | Sitting down to relax | Non-epistemic |
| 23 | Walking to move around | Non-epistemic |
| 24 | Running when in a hurry | Non-epistemic |
| 25 | Kicking with one's feet | Non-epistemic |
| 26 | Lifting objects with hands | Non-epistemic |
| 27 | Stretching one's muscles | Non-epistemic |
| 28 | Licking with one's tongue | Non-epistemic |
| 29 | Yawning when tired | Non-epistemic |
| 30 | Breathing heavily after exertion | Non-epistemic |
| 31 | Squatting down | Non-epistemic |
| 32 | Trembling at cold temperatures | Non-epistemic |
| 33 | Tickling a child to make them laugh | Non-epistemic |
| 34 | Touching other people with one's hands | Non-epistemic |
| 35 | Smelling the scent of food | Non-epistemic |
| 35 | Smelling the scent of food | Non-epistemic |
| 36 | Sleeping to restore one's energy | Non-epistemic |
| 38 | Sniffling when one has a cold | Non-epistemic |
| 39 | Crying at sad events | Non-epistemic |
| 40 | Dancing to a rhythm | Non-epistemic |
| 41 | Recalling past events | Epistemic |
| 42 | Judging one's options | Epistemic |
| 43 | Distinguishing between right and wrong | Epistemic |
| 44 | Reflecting on one's past and future | Epistemic |
| 45 | Having self-control of one's own behavior | Epistemic |
| 46 | Speculating about the possible outcomes of events | Epistemic |
| 47 | Making jokes | Epistemic |
| 48 | Thinking about magic | Epistemic |
| 49 | Using metaphors | Epistemic |
| 50 | Mourning the dead | Epistemic |
| 51 | Observing rituals | Epistemic |
| 52 | Overcoming a fear | Epistemic |
| 53 | Recognizing taboos | Epistemic |
| 54 | Recognizing relations among kin | Epistemic |
| 55 | Interpreting others' behavior | Epistemic |
| 56 | Symbolic reasoning | Epistemic |
| 57 | Making comparisons | Epistemic |
| 58 | Keeping track of time | Epistemic |
| 59 | Planning for the future | Epistemic |
| 60 | Recognizing melodies | Epistemic |
| 61 | Keeping track of people's age | Epistemic |
| 62 | Forming sentences | Epistemic |
| 63 | Forming words | Epistemic |
| 64 | Abstract reasoning | Epistemic |
| 65 | Having preferences concerning aesthetics | Epistemic |
| 66 | Attributing human qualities to inanimate objects | Epistemic |
| 67 | Having a belief in the supernatural | Epistemic |
| 68 | Having beliefs about fortune and misfortune | Epistemic |
| 69 | Devising classification of body parts | Epistemic |
| 70 | Having classification of animals | Epistemic |
| 71 | Having classification of plants | Epistemic |
| 72 | Having classification of weather | Epistemic |
| 73 | Devising methods to heal the sick | Epistemic |
| 74 | Having a contrast between "general" and "particular" | Epistemic |
| 75 | Having a logical notion of "and" | Epistemic |
| 76 | Having a logical notion of "not" | Epistemic |
| 77 | Forming myths | Epistemic |
| 78 | Having a concept of "person" | Epistemic |
| 79 | Having a preference for one's own children and close kin | Epistemic |
| 80 | Having norms about trade | Epistemic |

**Materials in Experiment 5**

Mary is a neuroscientist—she is one of the world’s leading experts on color vision. She knows exactly how color vision happens in the brain, and why we see color. For example, she understands what area of the brain encodes the color “red” (as distinct from “blue”). and how the neural firing gives rise to this specific percept. Mary fully understands how the brain gives rise to color vision.

Mary, however, has never seen color herself. She was brought up in a black-and-white room. Although she has seen many shades of black, gray, and white, Mary has never seen any other color.

So, Mary knows everything there is to know about how people see “red.” In particular, she understands how the brain interprets certain wavelengths as “red” and what physical and neural processes that make us see “red.” But she has never experienced how “red” feels.

Now, suppose Mary leaves her black-and-white room for the first time and sees a red rose. She watches the rose intensely, and registers its brightness and shine. Has she gained something new?

Jack is a professional billiards player—he is considered the world’s best player ever. Jack knows exactly how to launch a ball so it hits its target at the right angle and velocity. In the past ten years, he has won practically every game played. So clearly, Jack has an intimate and precise intuitive grasp of how billiard balls move.

 Jack, however, has no explicit understanding of the laws of physics. He has never finished high school. Although Jack constantly interacts with balls, he did not take a single physics course in his life.

So, Jack has perfect “gut” intuitions of how balls move. In particular, he can superbly predict their velocity and trajectory, and how their speed varies depending on the friction with the table. But Jack has never heard the term “momentum” or Newtonian physics.

Now, suppose that, on the advice of his agent, Jack takes a crash course in physics, where he learns the laws of motion, as they applied to launching billiard balls. Jack can now describe these laws perfectly. Has he gained something new?

Blix is an AI system that has acquired all this expert knowledge of vision neuroscience. Blix has all the information about how color vision happens in the brain, and why we see color. For example, Blix can indicate what area of the brain encodes the color “red” (as distinct from “blue”), and how the neural firing gives rise to this specific percept. So, Blix has a full command of how the brain gives rise to color vision.

Although Blix is an AI system, it does have a camera and sensors that allow it to encode color information. Blix, however, has never been exposed to colors; it has been built in a black-and-white room. Blix has encountered many shades of black, gray and white, but it has never encountered any other color.

So, Blix has command of everything there is to know about how people see “red.” In particular, it has all the information on how the brain interprets certain wavelengths as “red” and what physical and neural processes make us see “red.” But Blix has never encountered the color “red.”

Now, suppose Blix is presented with a red rose for the first time. Blix’s camera hovers over the rose for a while and registers the rose’s brightness and shine. Has Blix gained something new?

How much does this new encounter with a rose enrich its command of color vision?

Drox is an AI system that plays billiards—it is as good as the world’s best human player. Drox can launch a ball so it hits its target at the right angle and velocity. In the past ten years, he has won practically every game played. So clearly, Drox has a full grasp of how billiard balls move.

Although Drox is an AI system, it has no explicit grasp of the laws of physics. Its programmers have not input into it any physical laws; all its information was acquired by training it to move its artificial arms in the context of the billiard game.

So, Drox has perfect command of how billiard balls move. In particular, it can superbly predict their velocity and trajectory, and how their speed varies depending on the friction with the table. But Drox’s computer program has no terms like “momentum” or any laws of Newtonian physics.

Now, suppose an AI programmer decides to program into Drox some explicit laws of physics, including the laws of motion, as they apply to launching billiard balls. Drox can now describe these laws perfectly. Has Drox gained something new?

How much does the addition of these explicit physical laws enrich its command of the game?

1. I thank an anonymous reviewer for this summary of the argument. [↑](#footnote-ref-1)
